# Exoskeleton robot control for synchronous walking assistance in repetitive manual handling works based on dual unscented Kalman filter

**DOI:** 10.1371/journal.pone.0200193

**Published:** 2018-07-12

**Authors:** Fatai Sado, Hwa Jen Yap, Raja Ariffin Raja Ghazilla, Norhafizan Ahmad

**Affiliations:** Department of Mechanical Engineering, Faculty of Engineering, University of Malaya, Kuala Lumpur, Malaysia; Huazhong University of Science and Technology, CHINA

## Abstract

Prolong walking is a notable risk factor for work-related lower-limb disorders (WRLLD) in industries such as agriculture, construction, service profession, healthcare and retail works. It is one of the common causes of lower limb fatigue or muscular exhaustion leading to poor balance and fall. Exoskeleton technology is seen as a modern strategy to assist worker’s in these professions to minimize or eliminate the risk of WRLLDs. Exoskeleton has potentials to benefit workers in prolong walking (amongst others) by augmenting their strength, increasing their endurance, and minimizing high muscular activation, resulting in overall work efficiency and productivity. Controlling exoskeleton to achieve this purpose for able-bodied personnel without impeding their natural movement is, however, challenging. In this study, we propose a control strategy that integrates a Dual Unscented Kalman Filter (DUKF) for trajectory generation/prediction of the spatio-temporal features of human walking (i.e. joint position, and velocity, and acceleration) and an impedance cum supervisory controller to enable the exoskeleton to follow this trajectory to synchronize with the human walking. Experiment is conducted with four subjects carrying a load and walking at their normal speed- a typical scenario in industries. EMG signals taken at two muscles: Right Vastus Intermedius (on the thigh) and Right Gastrocnemius (on the calf) indicated reduction in muscular activation during the experiment. The results also show the ability of the control system to predict spatio-temporal features of the pilots’ walking and to enable the exoskeleton to move in concert with the pilot.

## Introduction

### Overview

Recently, research on powered wearable exoskeleton technology have been directed towards assisting workers in manual handling operations [1]. Manual handling operations involve repetitive or prolong static and dynamic body movements such as walking, standing, squatting, bending, twisting, etc. These works are notable causes of work-related musculoskeletal disorders (WRMSD) affecting all parts of the body. Manual-handling works in some industries like construction, agriculture, manufacturing, healthcare, hotels and retails involve prolong walking (and intermittent standing) which put the workers at high risk of work-related lower-limb disorders (WRLLD) [2]. Prolong walking can cause strain on the musculatures of the lower-limb, sprain on ligaments and tendons, and swollen legs [2, 3]. Severe health conditions such as osteoarthritis of the hip and knee, patellofemoral pain syndrome (PFPS), and immobility have also been reported [3, 4], which result in a number of sick leaves. Other, common consequences of prolong walking in the workplace are fatigue or muscular exhaustion leading to poor balance and fall [2].

**Fig 1:**
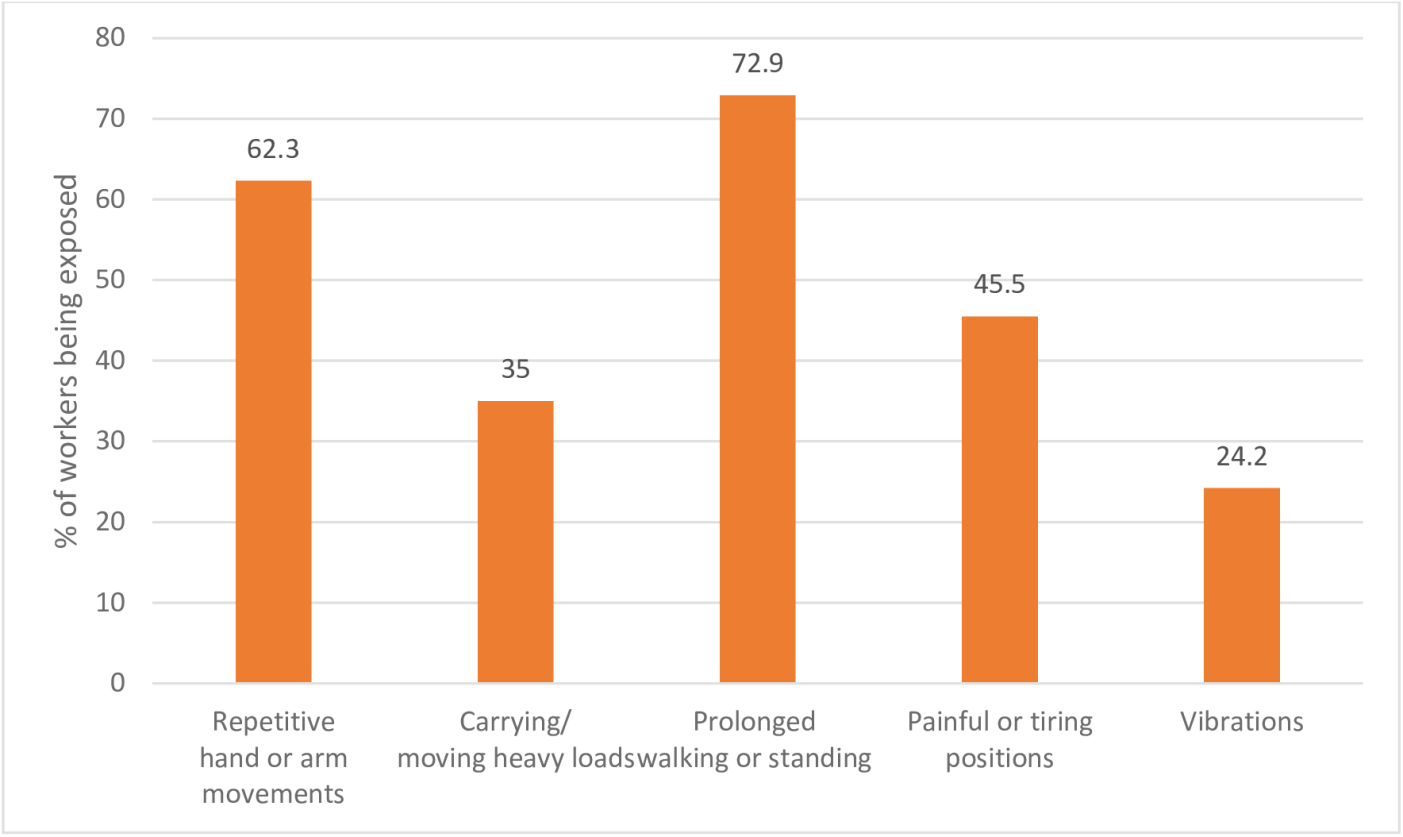
Risk factors for MSDs, percentage of workers being exposed a quarter of the working time in EU-27 member states [2].

Global data on work-related injuries due to prolong walking are sparse. Statistics from the EU nations and some Asian countries have however confirmed an increasing awareness of its high prevalence. A study among young workers in the manufacturing sector across 27 EU Member States indicated about 72.9% exposure rate to the risk of musculoskeletal disorder (MSD) due to prolong walking (or standing) [2] (see Fig 1). In France, the national SUMER survey revealed that about 74.9% of workers (in the surveyed industries) are frequently walking from one place to the other, which represent the highest risk factor of MSD [2]. Similar reports are found across the region. In Thai Public Hospital, a cross-sectional study conducted on 265 registered nurses associated prolonged walking to the risk of low back pain [5]. The conclusion is the same in another narrative survey carried out by Stolt, et al. [6] integrating data from several other countries across the globe.

Exoskeleton technology is a modern solution to assisting workers in manual handling jobs. Exoskeletons can improve workers efficiency, endurance and agility by augmenting the lower limb power, thus minimizing high muscular activation in prolong walking jobs. Deploying exoskeleton technology for human walking assistance, especially for able-bodied persons, is one of the challenging aspect of application of robotics [7, 8]. Human walking is dynamic and diverse, and for able-bodied persons, the exoskeleton needs capability to cope with this diversity for appropriate assistance. For obvious acceptability of exoskeleton suites in the industries, the exoskeleton should be capable of assisting its wearer to walk normally without impeding the wearer’s natural movement nor forcing the wearer into unintended motion [9]. A control technology that will enable the exoskeleton to learn or predict a wearer’s movement for synchronous walking assistance can be seen as one of the important requirement in exoskeleton technology for industrial manual handling applications [10–12].

### Related works

Several exoskeleton control techniques have been proposed for human walking assistance in the recent past. The techniques are often tailored to their intended application (and usually modified from a low-level position or force control). For medical and rehabilitation applications, there have been three popular techniques: the pre-define trajectory-based control technique, the assist-as-needed (AAN) control, and the intention-based control technique. These techniques have been applied to assist disabled (or neurologically impaired) patients and elderly subjects needing physical assistance for walking. For example, the pre-define trajectory based control [8]have been adopted to correct abnormal gait in patients with limb pathologies. The control technique mandates the subjects to follow a reference physiological gait trajectory (either similar to their own or) obtained from gait records of healthy subjects, which could normally contradict their own natural motion. Notable exoskeleton systems that have incorporated this technique for patients’ gait re-learning are the Lokomat [13] treadmill gait trainer, the ANdROS [14]gait rehabilitation system, the WalkTrainer [15] gait re-education system, and the Lower extremity powered system (LOPES) [16]. The assist-as-needed (AAN) control technique [17] on the other hand have been adopted to encourage subjects’ active participation in physical therapy. The technique is particularly useful at a later stage of patients’ gait rehabilitation to enhance motor function recovery. It enables exoskeleton assistance only when the subject is unable to carry-out the desired motion. AAN was applied in Active Assisted Leg (ALEX) exoskeleton [18]and LOPES [16]to assist only desired gait motion and to resist undesirable ones.

The third popular technique which can be refered to as intention-based control technique integrates sensors system (e.g. electromyogram (EMG) sensor, force/torque sensors, etc.) to the control framework to extract human intention for walking assistance or gait re-learning. Human intention based on EMG signal was extracted and used in HAL-3 [19, 20]exoskeleton system to assist the elderly and handicap in walking; and a 6-axis force/torque handle sensors was used in Intelligent Cane Robot [21]system to detect human intention for walking assistance.

These control techniques have been mainly developed for lower-limb disabled subjects with difficulty in walking (e.g. the elderly, the neurologically impaired, paraplegics, etc.). With regards to power or strength amplification applications for able-bodied walking assistance (such as industry workers engaged in prolong walking, or military personnel in long endurance trek, etc.), control requirement emphasizes natural (or synchronous) walking assistance since the able-bodied subjects can mainly walk normally on their specific gait. Four popular control techniques that tend to encourage natural walking or user-specific gait adaptation are identifiable from several reported studies. These include the sensitivity amplification control (SAC), the dynamic movement primitive, the adaptive frequency oscillator, and the EMG feedback control technique. For the purpose of this study, we provide some elaboration on these control techniques.

The sensitivity amplification control (SAC) was initially adopted for heavy load carrying while enabling the wearer to walk freely [22, 23]. SAC allows exoskeleton’s movement to overshadow wearer’s movements by increasing the closed loop system sensitivity to the wearers’ input force and torque, without any measurement from the wearer. Kazerooni, et al. [24] reported that SAC algorithm, implemented on BLEEX, could aid a wearer in carrying a payload (of 34kg) while walking on level ground at an average speed of 1.3m/s. The major limitation of SAC method however is its poor robustness to parameter variation. The method relies heavily on the accuracy of the exoskeleton model thus a slight parameter variation or disturbance can move the system to instability. Some improvements on SAC method using neural network algorithm have been recently reported by Long, et al. [25] and Yang, et al. [26].

Dynamic movement primitive is another strategy reported in literatures for walking assistance. DMPs are a set of nonlinear dynamic systems for generating discrete and periodic movement behaviors [27, 28]. They can be used as building blocks (similar to motor pattern generator (MPG) in neurobiology [29, 30] to generate/reproduce complex trajectories or control signals based on a learning signal (e.g. sensor signals). DMPs have been applied for imitation learning on humanoid robots [31] and for adaptive learning of joint torque profile in periodic task with an arm exoskeleton [32]. For able-bodied locomotion, Huang, et al. [33] reported an implementation of DMPs (combined with a locally weighted regression (LWR)) on a Human-powered Augmentation Lower Exoskeleton (HUALEX) system to learn and predict in real time the walking gait of human pilots. The authors indicated that HUALEX could follow pilots’ motion after one gait cycle’s correction. Standard DMPs are rich for encoding trajectories, however their main limitation is robustness to generate accurate movements in dynamic interaction situation. For improved performance, Gams, et al. [34] suggested an effective learning framework (e.g. LWR, reinforcement learning, etc.) that can deal with variable interaction dynamics from different wearers. A potential factor that also limit application of DMPs is the requirement of the knowledge of an appropriate -starting point- control policy (i.e. sets of nonlinear dynamic equations) that fit a particular movement behavior. This may be difficult to obtain except with expertise [28]. There is evidence of ongoing research with DMPs [35].

Adaptive frequency oscillator (AFO) is another powerful technique developed by Righetti, et al. [36] for reproducing cyclical movements e.g. periodic walking. AFO is basically a movement primitive with limit cycle attractor landscape [36, 37], capable of synchronizing with a periodic signal and extracting its features (like frequency, amplitude, envelop, etc.) in dedicated states variables [37]. AFOs have capabilities of predicting future joint positions of a pilot based on patterns learned during preceding cycles [38]. Just like DMPs, robotic assistance is then provided by attracting the subject’s joints to this future position using a force field [38], or a controller [39]. Lenzi, et al. [40] applied AFO on ALEX II exoskeleton [41] to assist human walking (to provide hip torque) and proved that the system could reduce hip muscle effort of the pilots. Ronsse, et al. [42] also applied adaptive oscillators to infer temporal derivatives (velocity and acceleration) of the human gait from noisy position signal of a pilot wearing LOPES lower-limb exoskeleton [16]. Traditional AFOs are mainly suitable for cyclical or periodic movements, which makes them less attractive for dynamic walking behavior typical in industrial operations. Matsubara, et al. [43] reported a simulation study to improve traditional AFOs by separating the gait pattern adaptation into style and phase parameters to account for diversity (style) in human walking. Practical implementation may still be required.

During walking, the muscles in the lower limb activates in a rhythmic pattern which corresponds to the walking pattern. The electrical activity, i.e. the electromyogram (EMG) signal, generated by the muscles therefore provides a basis for understanding or predicting a subject’s walking behavior or gait. This phenomenon has been exploited by a number of researchers to directly control lower-limb exoskeletons for walking assistance. Kawamoto, et al. [44] applied EMG feedback to control a HAL-3 robot to provide direct joint torque assistance (for walking) to subjects with gait disorder. Sawicki and Ferris [45] employed same proportional myoelectric control on a pneumatically powered ankle exoskeleton to provide ankle joint assistance with the aim to reduce metabolic cost of walking. Aguirre-Ollinger [46] adopt an indirect EMG feedback control approach by learning the muscle activation pattern via an AFO and thereafter apply the learned activation profile to provide direct torque command for the exoskeleton. EMG signal contains several information which however makes it difficult to precisely predict the motion of a pilot [47]. There are also inconsistencies in the estimated EMG signals from different subjects or from a particular subject [46, 47] in repeated trials since it is affected by a number of factors such as noise, location, placement of electrodes, user skin condition, etc.

### Our contributions

In this study, we propose a dual unscented Kalman filter (DUKF) to predict and generate human joint trajectories (spatio-temporal features of the human gait) based on a partial model of the coupled human-exoskeleton, and partial observation from noisy position sensors. We then attract the exoskeleton motion to this trajectory using an impedance controller integrated in a supervisory control architecture. The controller has capability to allow the exoskeleton to follow the human movement to enable synchronous dynamic walking, atypical in manual handling operations. The main motivation of this study is the need to provide lower-limb locomotion (walking) assistance to able-bodied subjects such as the manual-handling industry workers (or military personnel, nurses, etc.) who are routinely engaged in prolong walking. Exoskeleton technology can benefit these working personnel by reducing their lower-limb muscle activation or the onset of fatigue of the lower-limb (i.e. from the Quadriceps muscles, calf muscles etc.), and the risk of WRLLD due to prolong walking. Since exoskeleton assistance for able-bodied personnel needs to be flexible and natural (or synchronous) to facilitate dynamic walking, we propose the control technique based on Kalman filter that can predict/generate the human dynamic walking motion. The Kalman filter is popular in several fields including aerospace [48], SLAM [49] etc. for its accurate states prediction (or generation) in highly nonlinear, partially observed, dynamic systems, using its extended [50] or unscented versions [51]. It works fundamentally as a state observer, like the DMPs or AFOs, that can predict future estimates of states from current observations (poor or noisy signals from sensors). The dual estimation approach of Kalman filter exceptionally enhances its robustness to states prediction by concurrently estimating the model parameters (in a form of supervised learning approach) to improve state predictions. Thus, enhancing the overall system to model parameter variations such as can be found in dynamic (human-exoskeleton) walking situation.

The rest of the paper is organized as follows: Section II presents the modelling and description of the human-exoskeleton dynamic system. Section III introduces the design of the DUKF. Section IV presents the control architecture. Section V discusses the experiment design and result; and Section VI gives the discussion, conclusion, and recommendations.

## Human-exoskeleton system

### Human walking biomechanics

Knowledge of the biomechanics of the human walking is an important requisite for the development of exoskeletons. It is useful in planning appropriate torque and power requirements for exoskeleton drives and actuation systems. A number of literatures have given comprehensive analysis of the biomechanics of human walking [52, 53]. Two important aspect of the human biomechanics is the gait cycle and the joint kinematics and kinetics (i.e. joint angles, torques, and power).

#### Gait cycle

**Fig 2:**
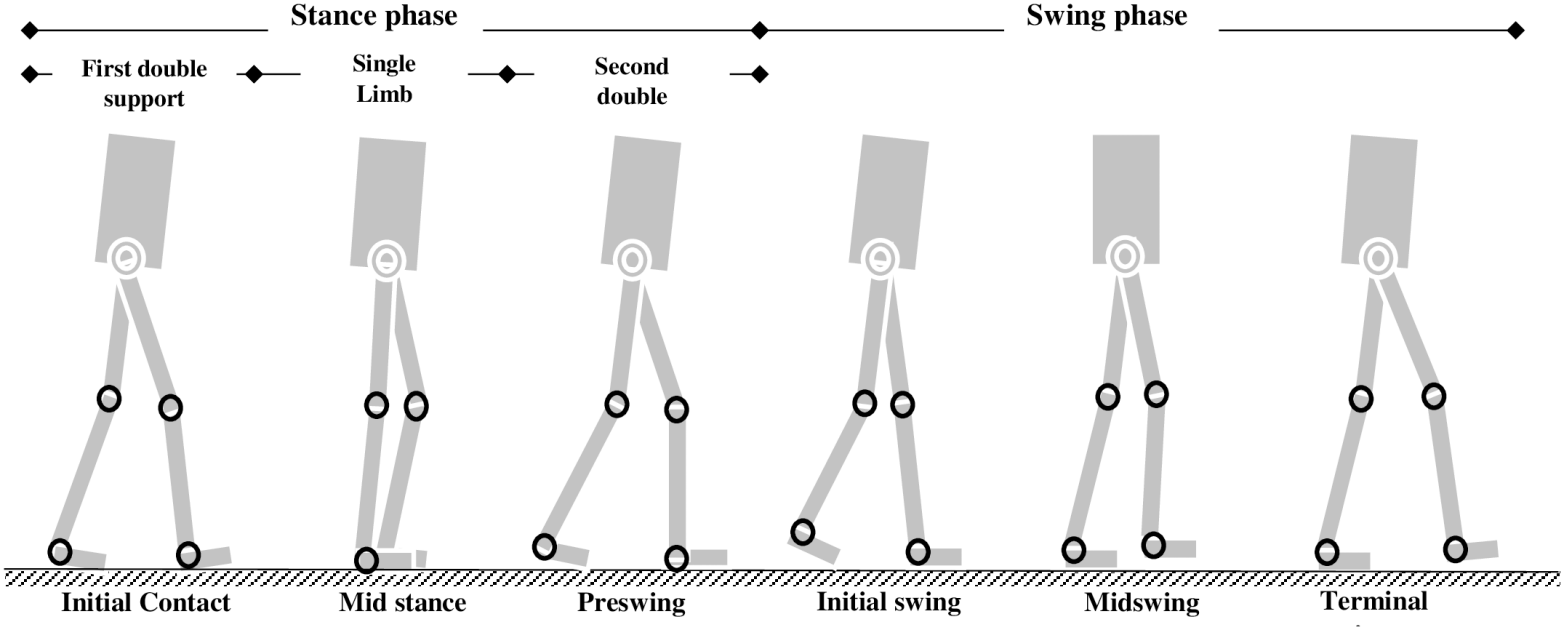
Walking phases.

Based on the duration between one heel strike (foot-strike) to the next heel-strike, the human walking is commonly divided into two cycles or phases: the stance and the swing (Fig 2). The stance phase is the foot-in-contact with ground duration. It dominates the walking by 62% whereas the swing phase- the foot off ground duration- takes the remaining 38%. The stance phase is also commonly divided into three stages [54]: initial double limb support (i.e. initial contact), single limb support (loading response, mid-stance, and terminal stance), and second double limb support (pre-swing). The swing phase on the other hand is sub-divided into three phases: initial swing (foot-off to foot clearance), mid-swing (foot clearance to tibia vertical) and terminal swing phase (tibia clearance to foot-strike).

During early stance, the hip extensors (hamstring muscle group etc.) contract to stabilize the hip while the quadriceps and tibialis anterior contracts eccentrically to support the heel strike. The ankle also dorsiflexes (tibialis anterior) eccentrically to control plantar flexion moment while the quads contract to stabilize the knee and counteract the flexion moment. At late stance, the toe flexors, the tibialis posterior, and the hip flexors (Quadriceps, sartorius, etc.) contract to propel the advancing limb. For the swing movement, the hip flexors contract concentrically to advance the swinging leg. This is followed by ankle dorsiflexion to ensure foot clearance (at mid-swing). Finally, in late swing, the hamstring muscles decelerate forward motion of the thigh preventing the leg from over-extending. The knowledge of the phases of human walking is extremely useful in developing exoskeleton control algorithms for human walking assistance.

#### Joint angles, torque, and power

The human body can be divided into three planes: sagittal, coronal (frontal), and horizontal (transverse) planes [8]. The dominant plane is the sagittal plane where most of the walking takes place. The other planes may be useful to study pathologies in walking or resolving balance problems [54]. Walking have a cyclic nature on the sagittal plane as can be seen from results of motion study in [52, 53]. The joint angles -hip, knee, and ankle joints- vary in quasi-sinusoidal manner. From a peak at heel strike (0% of gait cycle), a low at about mid-way (50%) of the cycle, to another peak at the next heel strike (100%). The flexion and extension range of motion of the hip joint on the sagittal plane can reach about 40 degrees for a normal adult male during level or loaded walking. The knee joint may extend up to 60 degrees, while the ankle dorsiflexes and plantarflexes over a relatively small range of about 29 degrees on the sagittal plane.

The torque and power profile on the hip, knee, and ankle are also quasi sinusoidal during level walking [52]. The three joints however exhibit unique kinetic properties for synergy in walking. The hip driving torque (≈0.6Nm/kg peak on sagittal plane [55]) and power is generated by tendon and muscle groups (gluteus maximus, psoas major, Iliacus, biceps femoris, etc.) connected at the hip and extending to the thigh. Hip torque is positive in the stance support phase as the hip support the stance leg and propel the leg forward [52, 56]. It goes negative, at terminal swing, as the tendons and muscles stretch to absorb energy, decelerating the leg prior to heel strike. The hip power exhibit both positive and negative peaks, but the average power transfer is found positive [56, 57]. This fact (positive and negative transfer of torque and power) suggests the prescription of bi-directional, back-drivable actuators when designing exoskeleton to assist hip sagittal motion. Hip abduction/adduction and internal/external motion on the coronal and transverse plane, on the other hand, are relatively small, thus can be assisted by passive elements like linear springs which can capture the negative power and release it during the positive driving phase for walking comfort [57].

The knee joint also exhibits positive energy phase (for torque generation) and a negative dissipative phase (for energy absorption). The average knee power (≈0.2Nm/kg peak torque on sagittal plane [55]) is however negative which indicates that the knee function more as a shock absorber dissipating energy during the walking cycle. During early stance, the knee transfers a region of negative energy to a region of positive energy (behaving like a spring), and thereafter it functions like a variable damper to control the leg in the swing motion [52]. The driving torque for flexing the knee joint is generated from the quadriceps femoris and sartorius. The extension is controlled by the hamstring muscles (e.g. biceps femoris, semitendinosus, and semimembranosus muscles). The behavior of the knee joint suggests the use of back-drivable actuators in conjunction with passive elements such as springs and dampers when designing knee joint of exoskeletons.

The ankle joint also exhibit positive and negative power transfer phase, however the average torque is almost entirely negative [52, 57]which indicate the functionality of the ankle as mainly dissipative absorbing the energy of walking. Under loaded walking, however, the ankle can generate more driving torque extending the phase of positive torque. For exoskeleton design, passive devices (spring and damper etc.) absorbing energy during negative power transfer and releasing it for positive work may be beneficial for ankle design of exoskeletons for walking assistance.

### Mechanical system

**Fig 3:**
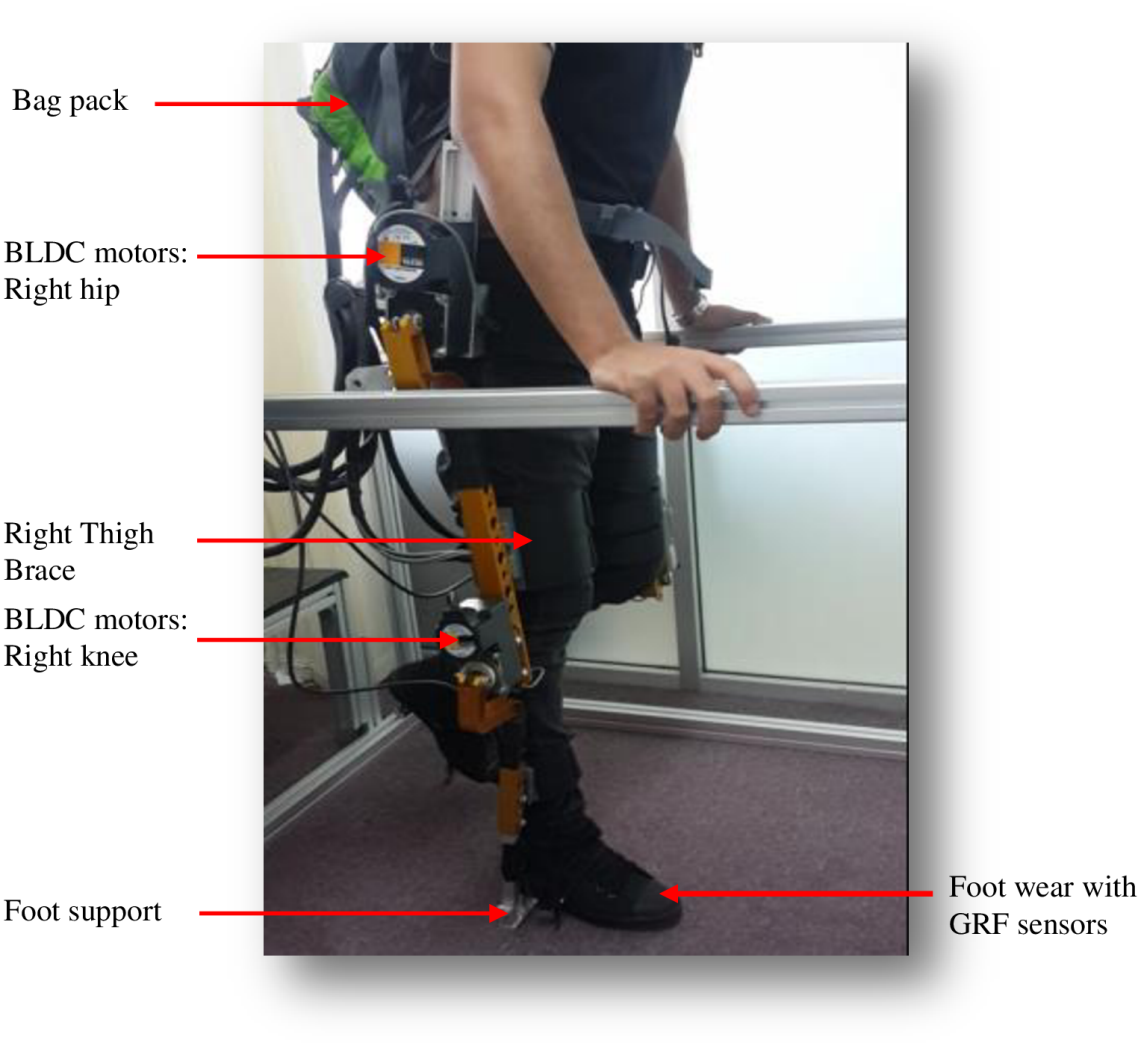
Exoskeleton prototype.

The prototype exoskeleton system (Fig 3) is a lower-limb anthropomorphic device with four degrees of freedom (DOF) on each leg: one active DOF at the hip and knee respectively, and 2 passive DOFs at the ankle for motion on the sagittal plane. The active hip and knee DOFs are actuated by back-drivable, bi-directional brushless DC motors with rated torques 38Nm and 17Nm respectively. The joints are equipped with sensors for torque and position measurement. The feet of the exoskeleton are designed into a dedicated pair of shoes for the pilot which enable the weight and bulk load of the exoskeleton to be directed to the ground. The insoles of the shoes are embedded with GRF sensors that are used to detect the pilot’s movement intention and phases of walking.

To ensure firm coupling between the pilot and the exoskeleton, there is provision of four soft braces attached at the thigh and shank links. These also facilitate compliance in coupled motion. Upper-body rigid support for the exoskeleton is partly provided by a backpack worn around the shoulder of the operator. Embedded in the backpack is a fairly thin aluminum plate attached on one end to a rigid mechanical bar around the hip, which serve as the torso of the exoskeleton.

The exoskeleton communication unit (i.e. the interface between the exoskeleton and the PC) is housed in the backpack to allow ease of movement. The communication unit consist of motor drivers, signal conditioning shields for the torque sensors, and a data acquisition system from National Instrument.

### Dynamic model of human-exoskeleton system

**Fig 4:**
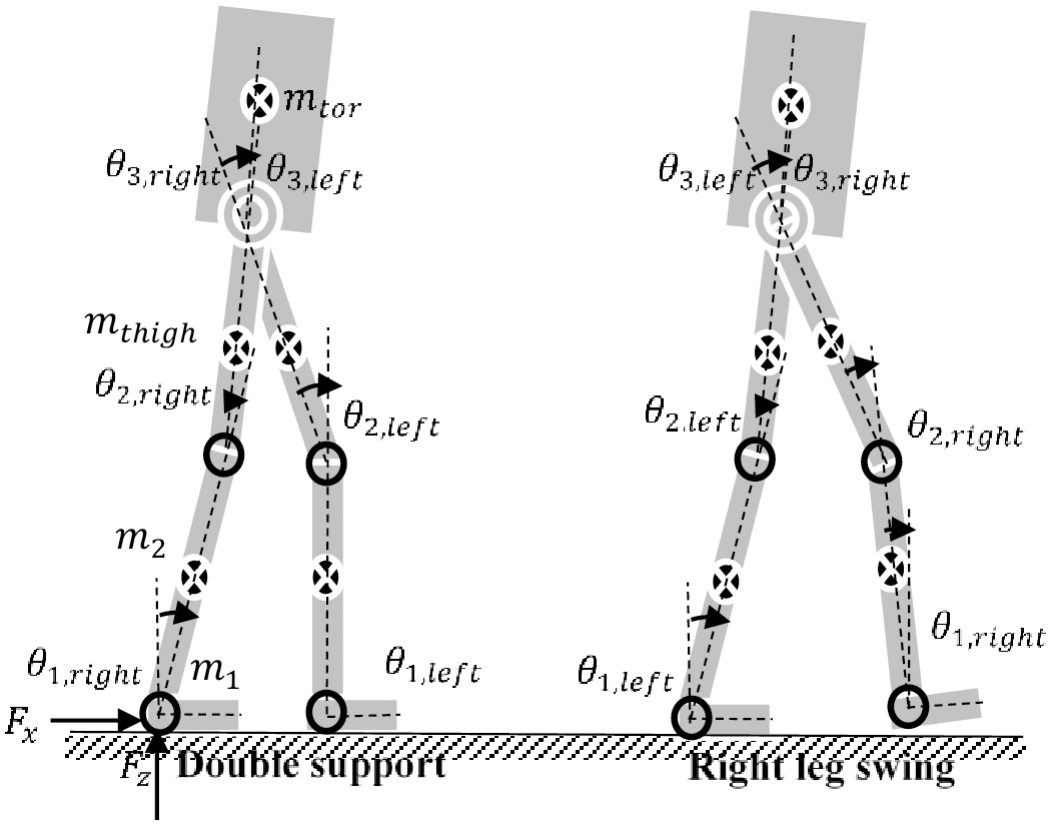
Schematic view of coupled human-exoskeleton walking motion in the sagittal plane.

The dynamic model of the coupled human-exoskeleton system for walking is derived by analyzing the kinematic configuration of the lower limb in each gait cycle. During the swing phase (single support phase), if a single leg model approach is adopted, it is possible to model the swing leg as a 3-DOF serial link mechanism pivoted to the hip joint and supporting only its weight, as adopted in the control of BLEEX [24]. For the stance leg, since it moves with a relatively small velocity and acceleration while carrying all the body weight, the effect of centripetal and Coriolis forces are minimal and therefore can permit flexibility in simplifying the dynamic model. It is possible to approximate it as a simple 1-DOF rigid link pivoting about the ankle joint as adopted in [58], however, in this study, we adopt a 3-DOF serial link model for both the swing and the stance leg of the coupled lower-limb human exoskeleton system (Fig 4).

We make some assumptions to obtain the partial model of the coupled system. The thigh, shank, and foot links of the exoskeleton are assumed lumped up firmly with corresponding segments of the human leg such that a new mass, and moment of inertia parameters can be obtained as approximate values. We also assume that their centers of mass are taken from the same references.

#### Swing leg

For the swing leg, the dynamics of coupled system for motion on the sagittal plane can be written in the general Euler-Lagrange form as:

$$\begin{array}{c} M\left(q\right)\ddot{q}+C\left(q,\dot{q}\right)\dot{q}+G\left(q\right)+\tau_{f}\left(q,\dot{q}\right)=\tau_{a}+\tau_{h} \end{array}$$ (1)

where *q* = [*θ*_1_, *θ*_2_, *θ*_3_]^*T*^ are the joint angles (Fig 4)and *M* is a 3 x 3 inertia matrix of the coupled human-exoskeleton leg and a function of *q*. *C* is a centripetal and Coriolis matrix and a function of *q* and $\dot{q}$. *G* is a 3 x 1 vector of gravitational torque and a function of *q*. *τ*_*f*_ is a vector of frictional torque. *τ*_*a*_ = [0, *τ*_2_, *τ*_3_]^*T*^ are the joints actuator torques respectively with the first element set to zero since there is no actuation for the ankle joint, *θ*_1_. *τ*_*h*_ is the 3 x 1 vector of joint human torque.

#### Stance leg

Similar to the model adopted in [23, 24], for stance phase, the dynamics of the prototype exoskeleton can be written as:

$$\begin{array}{c} M\left(q\right)\ddot{q}+C\left(q,\dot{q}\right)\dot{q}+G\left(q\right)+\tau_{f}\left(q,\dot{q}\right)=\tau_{a}+\tau_{h}-J^{T}F \end{array}$$ (2)

where all terms are as define in Eq (1), but this time, the torso mass *m*_*tor*_ is added to the thigh mass *m*_3_ = *m*_*thigh*_ + *m*_*tor*_. It affects the moment of inertia of the thigh link modifying the inertia matrix *M*, the centripetal and Coriolis matrix *C*, and the gravitational torque *G*. *F* is the 3x1 vector of ground reaction forces with Cartesian coordinates: *F*_*x*_, *F*_*y*_, and *F*_*z*_ (Fig 4); and *J* is the Jacobian matrix. Notice that, when the stance leg goes into swing in the next gait cycle, the torso mass no longer has influence on the swinging leg.

#### Joint friction and stiffness torque model

To minimize model uncertainties and to ensure an accurate relationship between the actuating torque and motion of the exoskeleton, an appropriate model of joint friction and stiffness torque is required. Our friction and stiffness torque model is given by [23, 59]:

$$\begin{array}{c} \tau_{f}\left(q,\dot{q}\right)=\left\{ \begin{array}{cc} b_{0} & q=0,\dot{q}=0\\ b_{1}sgn\left(\dot{q}\right)+b_{2}\left(\dot{q}\right) & \dot{q}\neq 0\\ \tau_{s}\left(q\right) & q\neq 0,\dot{q}=0 \end{array}\right. \end{array}$$ (3)

where *b*_0_ is the static friction torque; $b_{1}sgn\left(\dot{q}\right)$ is the coulomb or kinetic friction torque: a signed function of the joint angle velocity; and $b_{2}\left(\dot{q}\right)$ is the damping friction torque: a function of the joint velocity. *τ*_*s*_(*q*) is the stiffness torque which is a function of joint angle position. We present the estimation of the friction and stiffness torque parameters by a system identification method in the Result section.

#### Human-body segment mass

The human lower limb can be divided into six body segments: the right thigh (RT), the right calf/shank (RC), the right foot (RF), the left thigh (LT), the left calf/shank (LC), and the left foot (LF). The mass, centre of mass, and moment of inertia of these segments influence the dynamics of walking. They are important factors in estimating the joint driving torques and in deriving dynamic models of human walking. Techniques exist for estimating these segment parameters to some degree of accuracy. Based on cadaver averages, the segment masses can be estimated as a fraction of the body mass, while the segment centre of mass can be estimated as a fraction of their length to the proximal or distal end [52]. Geometric and anthropometric data from cadavers can also been used. The thigh and calf can be geometrically represented by cylinders, and the foot as a right pyramid [53]. Table 1 give the body segment parameters for the lower extremity of a normal adult male (as a fraction of the total body mass) estimated based on the technique of Vaughan, et al. [53]. The regression equations for estimating segment mass and moment is given as follows:

$$\begin{array}{c} Segment\;mass=C_{1}\left(Total\;body\;mass\right)+C_{2}{\left(Segment\;Length\right)}^{3}+C_{3} \end{array}$$ (4)

$$\begin{array}{c} Segment\;moment\;of\;inertia=C_{4}\left(Total\;body\;mass\right){\left(Length\right)}^{2}+C_{5} \end{array}$$ (5)

where *C*_1_, *C*_2_, *C*_3_, *C*_4_, and *C*_5_ are the regression coefficients.

**Table 1:** Body segment parameters of an adult male, adapted from [53].

	Body Segments					
Segment Parameter	RT	RC	RF	LT	LC	LF
Mass (kg/kg)	0.1057	0.0505	0.0119	0.1051	0.0505	0.0117
CoM (m/m)	0.39	0.42	0.44	0.39	0.42	0.44
Moment of Inertia (sagittal Flex/Ext)(Nm/Nm)	0.1238	0.0490	0.0035	0.1257	0.0490	0.0035

## The dual unscented Kalman filter design algorithm

The well-known Kalman filter is an optimal estimator for generating maximum-likelihood estimate of states of a linear, discrete-time, dynamic system [60]. It provides a more efficient recursive solution to the estimation problem in the sense that each updated estimate or prediction of states of a linear system is computed from previous estimate and new observation (sensor data), without need to compute estimates over an entire past observation data. It optimally combines noisy input observation with predictions from a known dynamic model.

**Fig 5:**
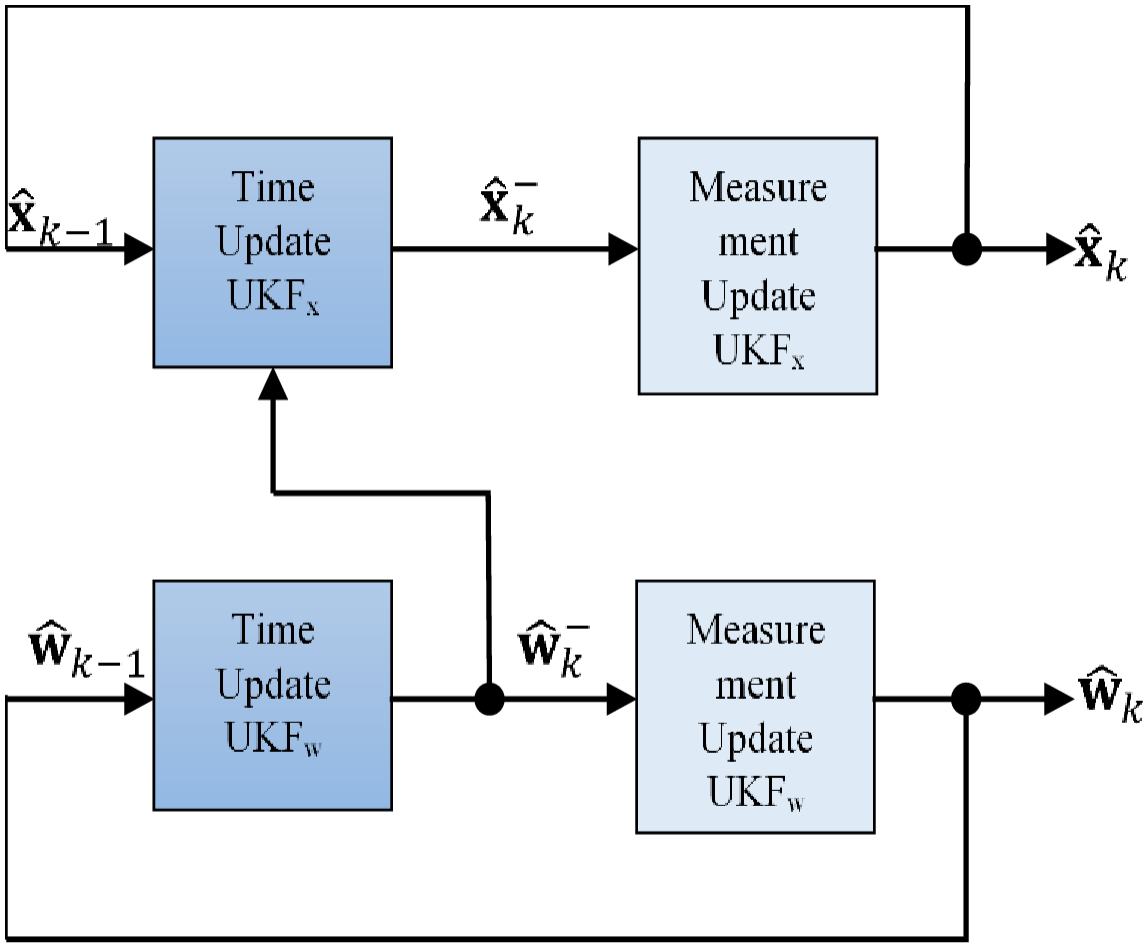
Dual estimation.

Aside states estimation, an important extension of the Kalman filter is for supervised learning or parameter identification of a partially known dynamic model given noisy observation. This important feature has motivated several applications in adaptive control involving the dual estimation of states and parameters. The dual estimation method works heuristically by alternating between estimate of states using the model, and estimate of the model using the states (Fig 5). If the model improves, so do the states.

The standard Kalman filter is however limited to linear systems. An extension to nonlinear systems necessitated the formulation of the Extended Kalman filter (EKF) [50] which involves first-order linearization of the nonlinear dynamic model. The linearization is done at every time step around the most recent estimate of states. The first order linearization and approximation by a Gaussian random variable GRV introduces large error in the true posterior mean and covariance of the transformed GRV which sometimes leads to divergence of the filter. A more superior approach to the EKF is the unscented Kalman filter (UKF) which solves the linearization problem using a deterministic sampling approach. UKF applies a minimal set of carefully chosen sample points, called sigma points, that completely capture the true posterior mean and covariance of the Gaussian random variable to a second order accuracy when propagated through the true non-linear system [51].

We apply the dual unscented Kalman filter approach in this study for dual estimation of hip and knee joints trajectories and model parameters update using the partial model of the coupled human-exoskeleton.

### States estimation

Consider the Exoskeleton dynamics given in Eqs (1) and (2), if transformed into stochastic non-linear states variable representation, we can write the non-linear state transition equation and measurement equation of the coupled system, respectively as

$$\begin{array}{c} x_{k+1}=F\left(x_{k},u_{k},w\right)+v_{k} \end{array}$$ (6)

$$\begin{array}{c} y_{k}=H\left(x_{k},n_{k}\right) \end{array}$$ (7)

where $x_{k}={\left[q^{T}\;{\dot{q}}^{T}\right]}^{T}$ denote the unobserved states of the system, and k∈[0,1,2,…∞] denotes discrete time. **y**_*k*_ represent the noisy position observation from sensors. **w** stands for the system parameters (e.g. link masses). **H** is the measurement function. **v** represents the process noise which is assumed additive with covariance matrix given as **P**_*v*_, and **n** denotes the measurement noise, non-additive, with covariance matrix given as **P**_*n*_. Both the process and measurement noise are assumed white and Gaussian, with zero mean (E[v]=E[n]=0).

The non-linear term F(•) is derived as

$$\begin{array}{c} F\left(•\right)=\left[\begin{array}{c} \dot{q}\\ -{\hat{M}}^{-1}\left(q,w\right)+\hat{C}\left(q,\dot{q},w\right)\dot{q}+\hat{G}\left(q,w\right)+{\hat{\tau }}_{f}\left(q,\dot{q}\right) \end{array}\right]+\left[\begin{array}{c} 0\\ -{\hat{M}}^{-1}\left(q,w\right) \end{array}\right]u \end{array}$$ (8)

where *u* = *τ*_*a*_ + *τ*_*h*_ − *J*^*T*^ *F*. The hat symbol ^ on the non-linear terms stands for the estimates. In the ensuing derivation, we adopt the notations used by Wan and Van Der Merwe [51].

Given the noisy observation **y**_*k*_ from sensor, at *k*^*th*^ sampling time, our goal is to generate optimal estimates of states, **x**_*k*_. The UKF does the estimation, much the same way as the EKF, using the following recursion [51]:

$$\begin{array}{c} {\hat{x}}_{k}=\left(prediction\;of\;x_{k}\right)+K_{k}\left[y_{k}-\left(prediction\;of\;y_{k}\right)\right] \end{array}$$ (9)

where ${\hat{x}}_{k}$ represents the optimal minimum mean-squared error (MMSE) estimate for **x**_*k*_ assuming that the prior estimate ${\hat{x}}_{k-1}$ and the current observation **y**_*k*_ are Gaussian Random Variables (GRV). If the (optimal) prediction of **x**_*k*_ is denoted ${\hat{x}}_{k}^{-}$, and the (optimal) prediction of **y**_*k*_ is denoted ${\hat{y}}_{k}^{-}$, their expectations can be expressed as:

$$\begin{array}{c} {\hat{x}}_{k}^{-}=E\left[F\left({\hat{x}}_{k-1},v_{k-1}\right)\right] \end{array}$$ (10)

$$\begin{array}{c} {\hat{y}}_{k}^{-}=E\left[H\left({\hat{x}}_{k}^{-},n_{k}\right)\right] \end{array}$$ (11)

where **v**_*k*−1_ (process noise) and **n**_*k*_(measurement noise) are also random variables (GRV). The optimal Kalman gain on the other hand is given by

$$\begin{array}{c} K_{k}=P_{x_{k}y_{k}}P_{{\tilde{y}}_{k}{\tilde{y}}_{k}}^{-1} \end{array}$$ (12)

It is expressed as a function of the posterior covariance matrices **P**_**x**_*k*_**y**_*k*__ and $P_{{\tilde{y}}_{k}{\tilde{y}}_{k}}$ (with ${\tilde{y}}_{k}=y_{k}-{\hat{y}}_{k}^{-})$ which also requires computation of the expectation of a nonlinear function of the prior state estimates. The UKF computes these expectations or optimal terms (${\hat{x}}_{k}^{-}$, ${\hat{y}}_{k}^{-}$, and **K**_*k*_) by generating a set of 2*L* + 1 *sigma* vectors **χ**_*k*−1_ (where *L* is the dimension of the state vector) symmetrically distributed around the prior (true) mean estimate ${\hat{x}}_{k-1}$.

$$\begin{array}{c} \chi_{k-1}=\left[{\hat{x}}_{k-1},\;{\hat{x}}_{k-1}+\sqrt{\left(L+\lambda \right)P_{k-1}},\;{\hat{x}}_{k-1}-\sqrt{\left(L+\lambda \right)P_{k-1}}\right] \end{array}$$ (13)

where, *λ* = *α*^2^(*L* + *κ*) − *L* is a composite scaling parameter. The constant *α* determines the spread of sigma points around ${\hat{x}}_{k-1}$, and is usually set to a small positive value in the range 1*e*^−3^. The constant *κ* is a secondary scaling factor, which is usually set to zero (or 3 − *L*). $P_{k-1}=E\left[\left(x_{k-1}-{\hat{x}}_{k-1}\right){\left(x_{k-1}-{\hat{x}}_{k-1}\right)}^{T}\right]$ is the state covariance matrix.

The generated sigma points **χ**_*k*−1_ are propagated through the non-linear system in Eqs (6) and (7), see Eqs (14)–(16), to obtain the optimal predictions ${\hat{x}}_{k}^{-}$, ${\hat{y}}_{k}^{-}$ and the prior covariance ${\hat{P}}_{k}^{-}$ for the recursion in Eq (9) to a 3rd order accuracy (of the Taylor series expansion), see Eqs (17)–(19).

$$\begin{array}{c} \chi_{\left(k|k-1\right)}^{*}=F\left(\chi_{k-1},u_{k-1},w\right) \end{array}$$ (14)

$$\begin{array}{c} \chi_{\left(k|k-1\right)}=\left[\chi_{\left(0,k|k-1\right)}^{*},\;\chi_{\left(0,k|k-1\right)}^{*}+\gamma \sqrt{P_{v}},\;\chi_{\left(0,k|k-1\right)}^{*}-\gamma \sqrt{P_{v}}\right] \end{array}$$ (15)

$$\begin{array}{c} y_{\left(k|k-1\right)}=H\left(\chi_{k|k-1}\right) \end{array}$$ (16)

$$\begin{array}{c} {\hat{x}}_{k}^{-}=\sum_{i=0}^{2L}W_{i}^{\left(m\right)}\chi_{\left(i,k|k-1\right)}^{*} \end{array}$$ (17)

$$\begin{array}{c} {\hat{P}}_{k}^{-}=\sum_{i=0}^{2L}W_{i}^{\left(c\right)}\left(\chi_{\left(i,k|k-1\right)}^{*}-{\hat{x}}_{k}^{-}\right){\left(\chi_{\left(i,k|k-1\right)}^{*}-{\hat{x}}_{k}^{-}\right)}^{T}+P_{v} \end{array}$$ (18)

$$\begin{array}{c} {\hat{y}}_{k}^{-}=\sum_{i=0}^{2L}W_{i}^{\left(m\right)}y_{\left(i,k|k-1\right)} \end{array}$$ (19)

where $W_{i}^{\left(•\right)}$ are the generated weights alongside the sigma points given by

$$\begin{array}{c} W_{i}^{\left(m\right)}=W_{i}^{\left(c\right)}=\frac{\lambda }{\left(2(L+\lambda )\right)},\;\;i=1,\ldots ,2L \end{array}$$ (20)

with initial values, $W_{0}^{\left(m\right)}=\frac{\lambda }{\left(L+\lambda \right)}$, and $W_{0}^{\left(c\right)}=\frac{\lambda }{\left(L+\lambda \right)}+1-\alpha^{2}+\beta$. The constant *β* is used to incorporate prior knowledge of the distribution. For Gaussian distribution, *β* = 2 is optimal [51]. Notice that the superscripts (*m*) and (*c*) implies weighting factors for states and covariances respectively. The terms $\chi_{\left(i,k|k-1\right)}^{*}$ (augmented as **χ**_(*i*, *k*∣*k*−1)_), and **y**_(*i*, *k*∣*k*−1)_ denote the posterior (propagated) sigma vectors of the process and observation functions respectively.

The posterior covariances for computing the optimal gain are thus given as

$$\begin{array}{c} P_{{\tilde{y}}_{k}{\tilde{y}}_{k}}=\sum_{i=0}^{2L}W_{i}^{\left(c\right)}\left(y_{\left(i,k|k-1\right)}-{\hat{y}}_{k}^{-}\right){\left(y_{\left(i,k|k-1\right)}-{\hat{y}}_{k}^{-}\right)}^{T} \end{array}$$ (21)

$$\begin{array}{c} P_{x_{k}y_{k}}=\sum_{i=0}^{2L}W_{i}^{\left(c\right)}\left(\chi_{\left(i,k|k-1\right)}-{\hat{x}}_{k}^{-}\right){\left(y_{\left(i,k|k-1\right)}-{\hat{y}}_{k}^{-}\right)}^{T} \end{array}$$ (22)

Eqs (17), (19), (21) and (22) are used in the recursion (during the measurement update phase) to generate the optimal estimate of states **x**_*k*_. Details of the UKF procedure for states estimation/generation can be found in [51].

### Parameter estimation

For system identification of the parameters of the model in Eqs (1) and (2), we define a new state-space formulation given as

$$\begin{array}{c} w_{k+1}=w_{k}+r_{k} \end{array}$$ (23)

$$\begin{array}{c} d_{k}=Y\left(x_{k},w_{k}\right)+e_{k} \end{array}$$ (24)

where $w_{k}={\left[m_{1}^{T},m_{2}^{T},m_{3}^{T}\right]}^{T}$ corresponds to the partially known model parameters: represented here as a stationary process with identity state transition matrix, driven by the process noise **r**_*k*_ with covariance $E\left[r_{k}r_{k}^{T}\right]=P_{r_{k}}$. The parameters *m*_1_, *m*_2_, and *m*_3_ represent the link masses (Fig 4). The model parameters **w**_*k*_ are assumed constant only perturbed by the process noise. The output **d**_*k*_ corresponds to a nonlinear observation on **w**_*k*_(in this case, a torque output); and **e**_*k*_ corresponds to the error in the non-linear model. We define Y(•) from Eqs (1) and (2) as

$$\begin{array}{c} M\left(•\right)\ddot{q}+C\left(•\right)\dot{q}+G\left(•\right)+\tau_{f}\left(•\right)=Y\left(•\right) \end{array}$$ (25)

The UKF also estimates the model parameters **w**_*k*_ using the recursion given in Eq (9) and by propagating a set of generated sigma points. As an optimization approach, the UKF attempt to minimize the prediction error cost on every time step (similar to the EKF) using the cost function given by

$$\begin{array}{c} J\left(w\right)=\sum_{t=1}^{k}{\left[d_{t}-Y\left(x_{t},w\right)\right]}^{T}P_{e_{k}}^{-1}\left[d_{t}-Y\left(x_{t},w\right)\right] \end{array}$$ (26)

where **P**_**e**_*k*__ is the estimation error covariance $E[e_{k}e_{k}^{T}]$. In this study we chose the innovation covariance **P**_**r**_*k*__ based on the recursive least square algorithm [61] define as:

$$\begin{array}{c} P_{r_{k}}=\left(\lambda_{RLS}^{-1}-1\right)P_{w_{k}} \end{array}$$ (27)

where $\lambda_{RLS}\in \left(0,1\right)$ is the forgetting factor. The rate of convergence and tracking performance of the UKF filter is influenced by the innovation covariance **P**_**r**_*k*__. The constant *λ*_*RLS*_ provides an exponentially weighting on past data which makes it possible to emphasize the most recent data. Notice that this feature is useful to enable tracking of the complex motion dynamics of human walking.

## The control architecture

In the preceding section, we presented the method of trajectory generation and prediction of the human walking based on dual unscented Kalman filter. The only input to the filter is the noisy position data from sensor and the output is the predicted joint kinematics, ${\hat{x}}_{k}$ (i.e. joint spatio-temporal variables). In this section, we introduce an impedance control law under a supervisory controller to achieve reference tracking of the generated trajectory and coupled interaction control with regards the human walking biomechanics.

### Impedance control

Given the exoskeleton dynamics in Eqs (1) and (2), we define the impedance based computed torque control law, *τ*_*c*_

$$\begin{array}{c} \tau_{c}=M\left(q\right)u_{a,q}+C\left(q,\dot{q}\right)\dot{q}+G\left(q\right)-\tau_{h} \end{array}$$ (28)

where,

$$\begin{array}{c} u_{a,q}={\ddot{q}}_{d}+M_{r}^{-1}\left[B_{r}\left({\dot{q}}_{d}-\dot{q}\right)+K_{r}\left(q_{d}-q\right)\right] \end{array}$$ (29)

The variables *q*_*d*_, ${\dot{q}}_{d}$, and ${\ddot{q}}_{d}$ are the reference joint kinematics: 3x1 vectors of joint position, velocity and acceleration respectively which are predicted from the DUKF. The constants *M*_*r*_, *B*_*r*_, and *K*_*r*_ are the controller impedance parameters (inertia, viscous damping, and stiffness parameters respectively).

### Supervisory control

**Fig 6:**
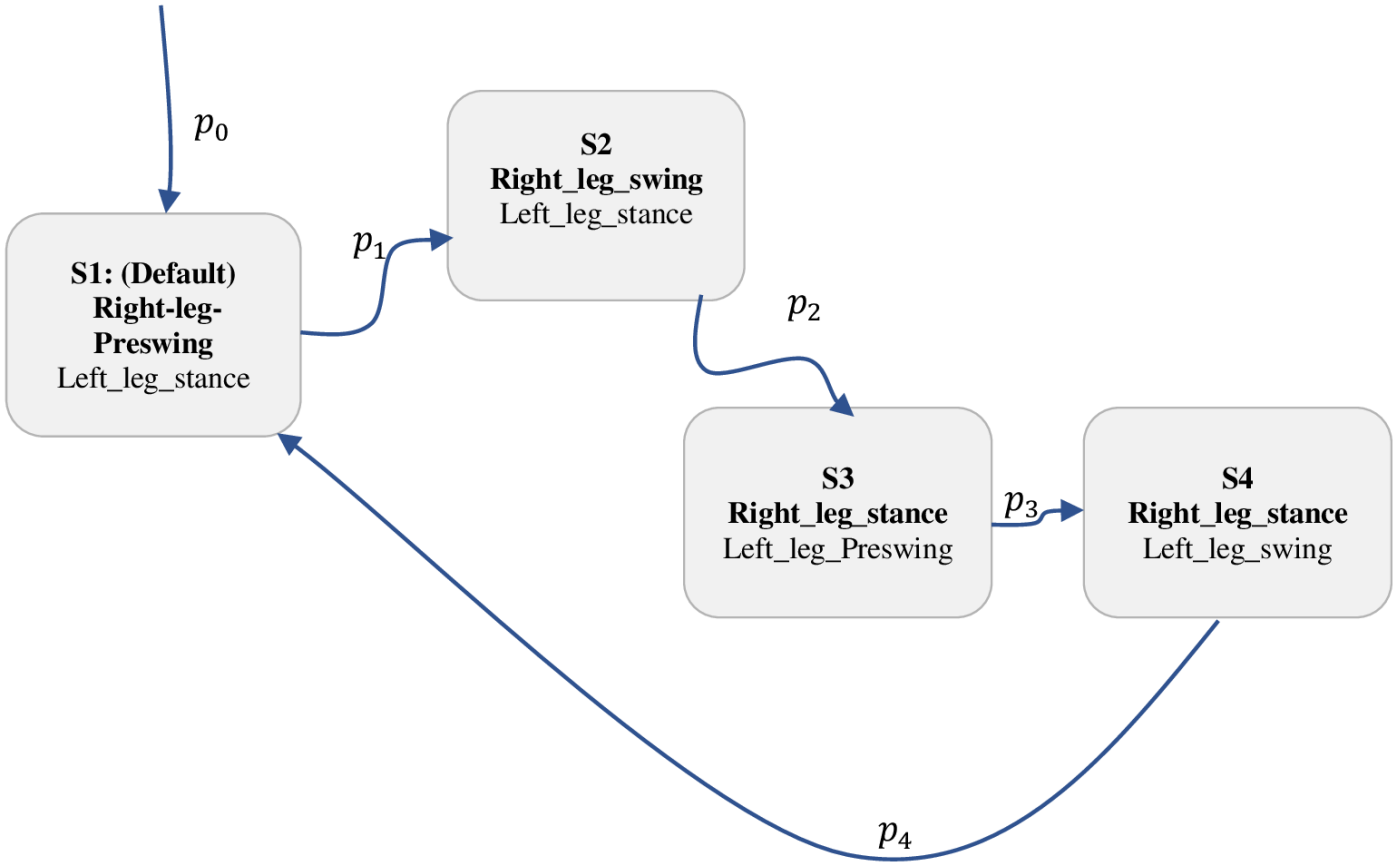
4-state hybrid automaton for swing to stance transition.

To synchronise the movement of the exoskeleton with the pilot motion, we developed an outer-loop supervisory controller. The supervisory controller implements the intention detection system and gait phase detection in a 4-state hybrid automaton (Fig 6). Three events are used to detect the pilot’s intention and to enable transition between the swing and the stance phase: the heel-off, the heel-strike, and heel-flat. These events are captured by the insole ground reaction force sensors (GRF). The heel-off event detects the pilot intention to initiate swing motion while the heel-strike gives indication of the beginning of stance phase. The heel-flat event (at mid-stance) for one leg allows the alternate leg to move from pre-swing to swing, and/or from swing to heel-strike. The automaton captures a gait cycle as the period between one heel-off event to the next, or heel-strike to the next, while the switching from swing to stance mode is indicated by the alternation from heel-off to heel-strike. For the purpose of switching the hybrid automaton or detecting the events, we compute a fractional index, *P*, of the ground reaction force for each leg as

$$\begin{array}{c} P=\frac{\alpha_{1}F_{1}+\alpha_{2}F_{2}}{F_{1}+F_{2}} \end{array}$$ (30)

where *F*_1_ is the force measure from the insole GRF sensor placed at the heel (rear), and *F*_2_ is the force measure from the sensor placed at the ball of foot (front). The constants *α*_1_ = 1, and *α*_2_ = −1 are chosen arbitrary, thus *P* is computed in the range [-1, 1]. An approximate positive *P* value of 1 (P≈1) indicates a heel-strike, while an approximate negative P value of -1 (P≈-1) indicates heel-off. An approximate zero value of *P* (P≈0) gives the indication of heel-flat (mid-stance).

**Fig 7:**
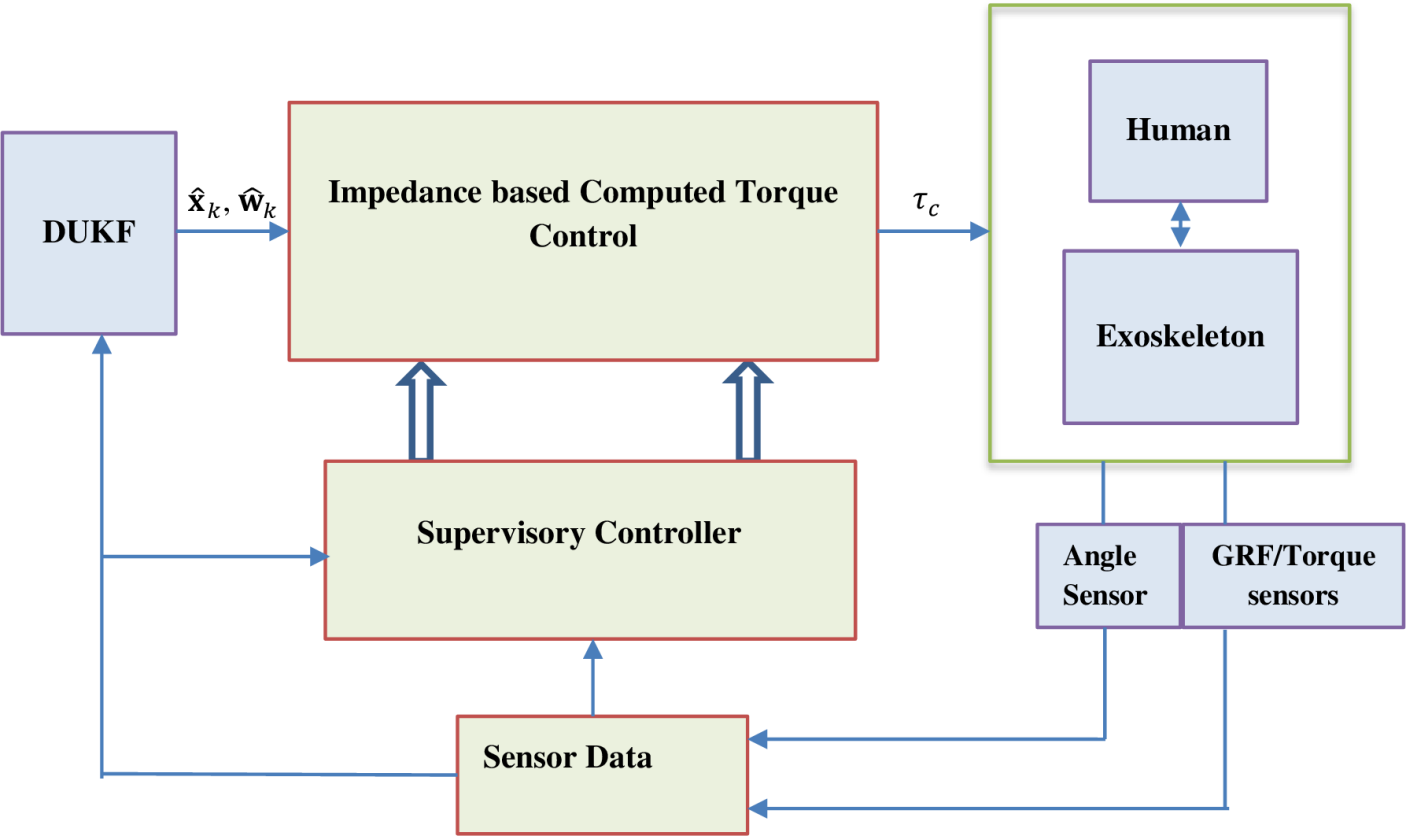
Control architecture.

Based on the computed *P* value (for the left and right leg), the 4-state hybrid automaton is transited to coordinate and synchronize the motion of the left and right leg. Each state thus enables the trajectories of the hips and knees. Table 2 gives summary states transition logic of the finite state automaton and Table 3 presents the events logic. Fig 7 shows the overall controller architecture.

**Table 2:** States transition logic.

	Trajectories			
States	Right Leg	Mode	Left leg	Mode
S1	Right-leg-Preswing	Swing	left-leg-stance	Stance
S2	Right-leg-swing	Swing	Left-leg-stance	Stance
S3	Right-leg-stance	Stance	Left-leg-Preswing	Swing
S4	Right-leg-stance	Stance	Left-leg-swing	Swing

**Table 3:** Events logic.

Events		
Right Leg	Left leg	Transition (*P*)
Default (initial position)	Default (initial position)	*p*_0_
Heel-off (P≈-1)	Heel-flat (P≈0)	*p*_1_
Heel-strike (P≈1)	Heel-flat (P≈0)	*p*_2_
Heel-flat (P≈0)	Heel-off (P≈-1)	*p*_3_
Heel-flat (P≈0)	Heel-strike (P≈1)	*p*_4_

## Experimental setup

In this section, we present the experimental setup and evaluation of the control strategy on the exoskeleton system. Three aspects of the system are evaluated: the capability of the system to minimize subjects’ lower limb muscle activation during walking, the capability to predict subjects’ gait trajectory, and the controller performance for reference tracking.

### Ethics clearance

Participants gave their written informed consent to participate in the experimental study. The ethics clearance for the experiment was granted by the University of Malaya Research Ethics Committee (UMREC).

**Fig 8:**
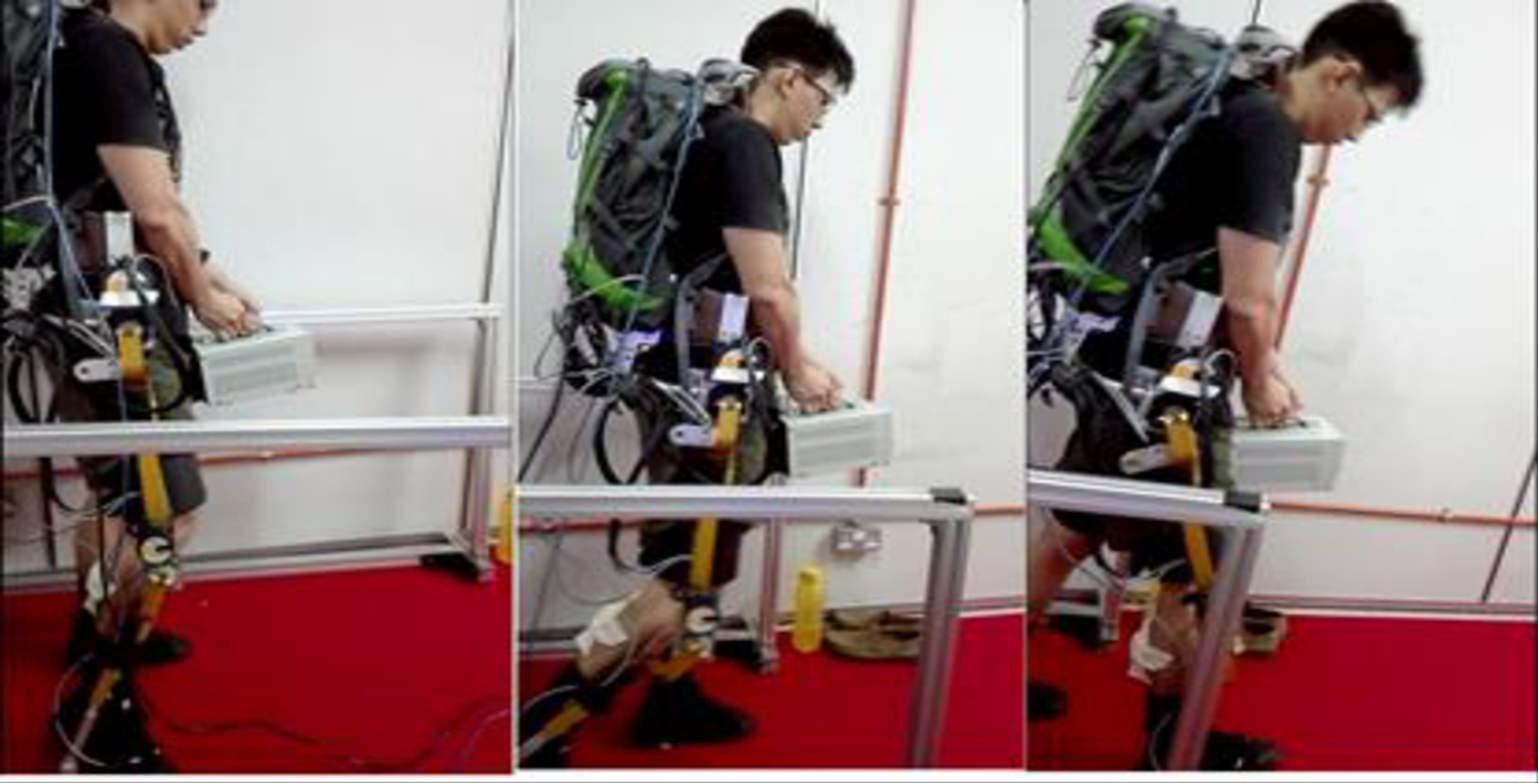
Load carrying operation within 10m walking distance by one of the participants.

Note: the individual in this manuscript (Fig 8) has given written informed consent (as outlined in PLOS consent form) to publish these case details.

### Participants

Four subjects with average age of 25 ± 5 years, average height of 169 cm ± 2cm and average weight of 77kg ± 7kg participated in the experiment. Two of the subjects were recruited in September, 2017 from the Centre of Product Design and Manufacturing (CPDM), Department of Mechanical Engineering and the other two subjects were recruited in April, 2018 from the Mechanical Engineering Workshop, Department of Mechanical Engineering. Initially five subjects from the Mechanical Engineering Workshop consented to participate in the experiment, however, three drop-out and only two were available for the experiment. All the subjects who participated have no history of musculoskeletal disorders or neurological diseases. The physiological features of the participants such as height were considered in their selection to ensure fitness with the exoskeleton suite.

### Experimental design procedure

The participants were guided generally on the functionalities of the wearable exoskeleton suit and how to use the power-down switch to shut-down the system for safety in case of discomfort and emergency. Preliminary tests were also conducted to further acquaint the participants with the operation of the exoskeleton. One EMG sensor from Shimmer Sensing Technology is firmly attached at the location of the Right Vastus Intermedius (Quadriceps muscles on the right thigh) and another one attached at the location of the Right Gastrocnemius (calf muscles on the right leg) to monitor/record each participants’ muscle activity level during walking. MATLAB software is used for rectification, low pass filtering, and analysis of the recorded EMG signals. Fig 8 shows participant 1 undergoing walking and carrying experiment.

The task of the experiment was to carry an object/load weighing 2.0kg while walking on horizontal ground a distance of 10m in three different modes: load carrying without wearing the suite, load carrying with suite in passive mode, and load carrying with suite in active mode.

#### Mode 1: Load carrying without suite

In this mode, the participants are to carry the load of mass to the stated distance, walking freely without wearing the exoskeleton suite. This procedure was repeated three times. The subjects were instructed to walk at their normal speed levels with a 2 minutes rest interval between each trial. The subjects’ EMG signals from the two lower limb muscles were recorded to evaluate subjects’ average muscle activation level during load carrying and walking.

#### Mode 2: Load carrying with suite in passive mode

The second mode was carried out mainly to compare the lower limb muscle activation level when carrying the same load, walking, fully-dressed, on the exoskeleton suit. The same procedure in the first was also repeated. The exoskeleton is operated in passive mode (unactuated) in this procedure. The driving torque to move exoskeleton thus come from the wearer.

#### Mode 3: Load carrying with suite in active mode

In this mode, the participants repeated the same procedure in Mode 2 but with active motor assistance from the exoskeleton. The muscle activity on the two lower limb muscles and the controller performance for trajectory generation, tracking and parameter estimation were evaluated.

## Results

### Muscle activity

**Fig 9:**
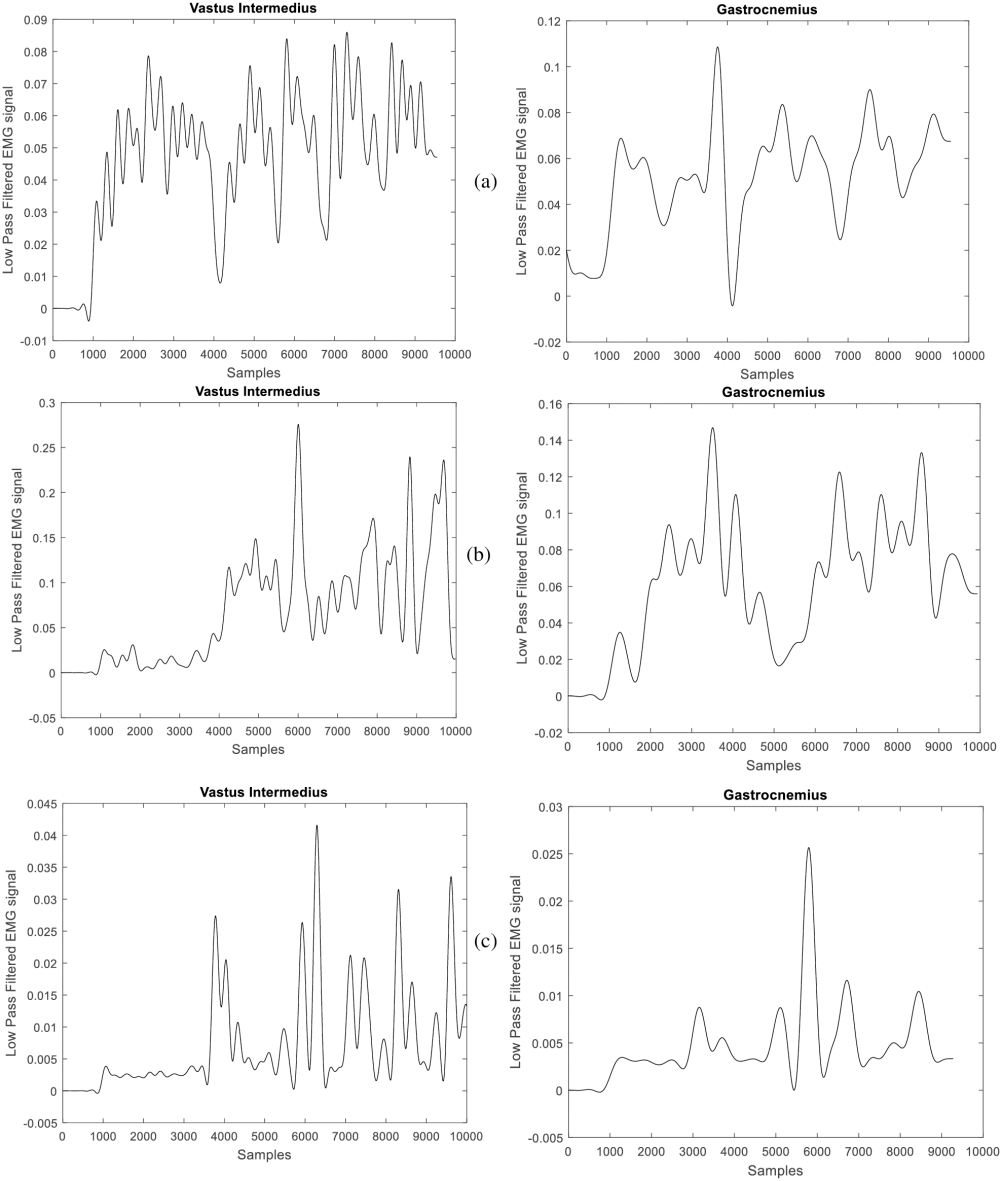
Lowpass filtered EMG signals recorded at the right vastus intermedius and right Gastrocnemius of participant 1. (a) free walking experiment, (b) walking in passive exoskeleton mode and (c) walking in active exoskeleton mode.

**Fig 10:**
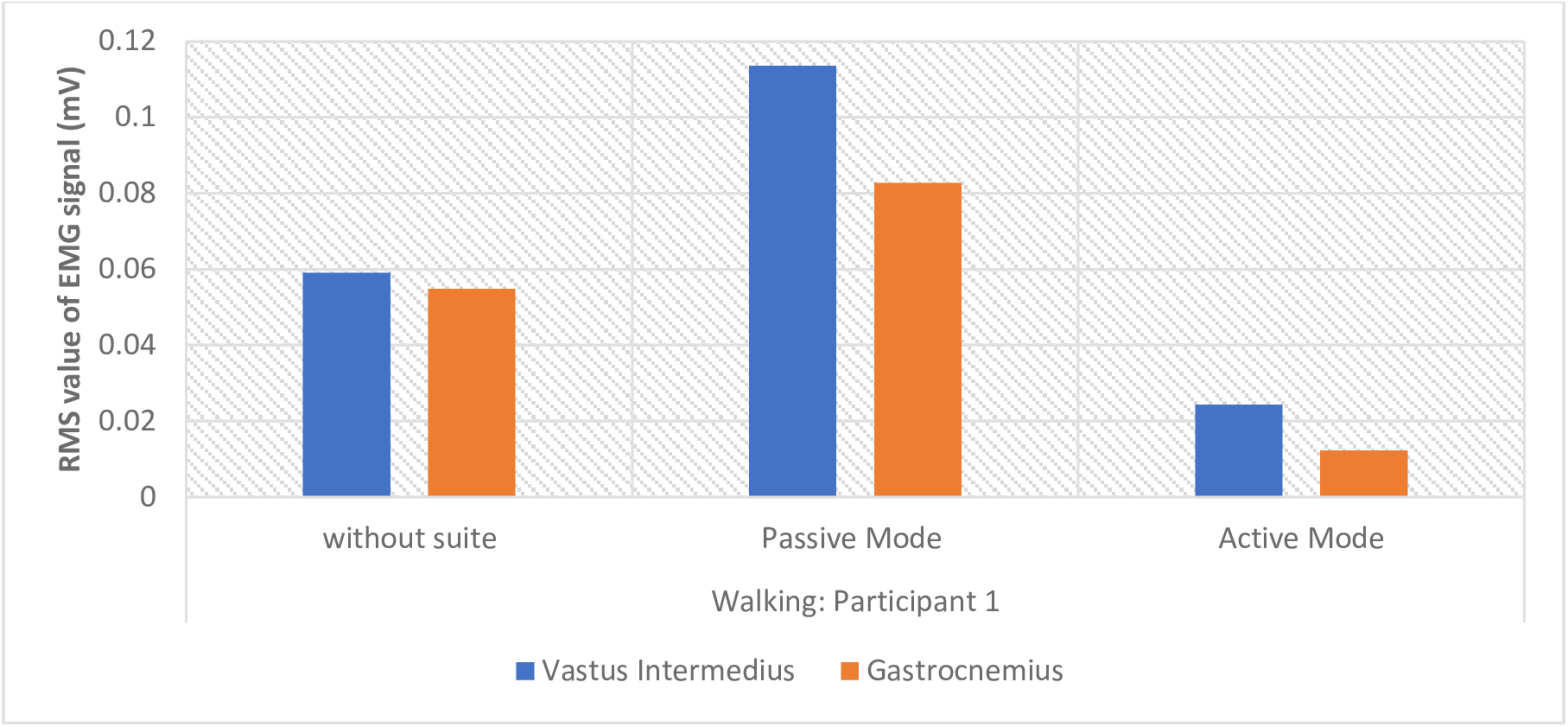
Bar chart showing mean muscle activation levels of one participant during walking movements.

The lowpass filtered EMG signals taken from the 1st participant’s Right Vastus Intermedius and the Right Gastrocnemius for the three modes: carrying a load while walking freely (without suite), walking in passive mode with suite, and walking in active mode with suite are shown in Fig 9. We present the muscle activity plot for one participant for the sake of brevity. Generally, the analysis of result is to compare the mean values of the root mean square (RMS) of the EMG signals (which give the measure of the amount of muscle activity) recorded for each participant in the three separate modes of performing the task. Since we performed repeated trials, a one-way ANOVA with repeated measures was adopted to determine the mean RMS value for each task mode and to determine whether there is any statistical significance between these means. Summary of findings is presented in Table 4. Pairwise Comparisons of the differences between the means is computed based on estimated marginal means, at 95% confidence interval for difference, see Table 5. Table 6 gives the percentage reduction in muscle activity for each participant computed from the differences in mean between the active mode and free walking mode. We define statistical parameters as: **M** is mean, **MD** is mean difference, **SD** is standard deviation from the mean, **SE** is standard error of the mean, and **p** is the confidence level. The average recorded muscle activity, for all the participant, during the active mode experiment (Right Vastus intermedius- **M** = 0.0250mV, **SD** = 0.011mV; and Right Gastrocnemius- **M** = 0.0173mV, **SD** = 0.00667) was found to be significantly less than the average muscle activity recorded during the free walking mode (Right Vastus intermedius: **M** = 0.0465mV, **SD** = 0.0220mV; and Right Gastrocnemius: **M** = 0.0447mV, **SD** = 0.0121). The highest muscle activity is recorded during the passive mode experiment (Right Vastus intermedius: **M** = 0.0842mV, **SD** = 0.0421mV; and Right Gastrocnemius: **M** = 0.0782mV, **SD** = 0.0151). The bar chart in Fig 10 shows the comparison plot of muscle activity for the three modes, for participant 1.

**Table 4:** Recorded EMG signal of four participant taken over three trials.

Muscles	Vastus-Intermedius RMS (mV)		Gastrocnemius RMS (mV)	
**Participant 1**	**M**	**SD**	**M**	**SD**
Free walking	0.0591	0.0101	0.0548	0.00477
Suite in passive mode	0.1136	0.0147	0.0827	0.00881
Suite in active mode	0.0243	0.00514	0.0123	0.00237
**Participant 2**	**M**	**SD**	**M**	**SD**
Free walking	0.0722	0.00537	0.0378	0.00568
Suite in passive mode	0.1193	0.02108	0.0919	0.00525
Suite in active mode	0.0291	0.00969	0.0135	0.00230
**Participant 3**	**M**	**SD**	**M**	**SD**
Free walking	0.0313	0.00789	0.0434	0.0148
in passive mode	0.0631	0.03477	0.0635	0.0174
Suite in active mode	0.0190	0.01064	0.0250	0.00384
**Participant 4**	**M**	**SD**	**M**	**SD**
Free walking	0.0276	0.00981	0.0429	0.01714
Suite in passive mode	0.0406	0.03516	0.0748	0.01365
Suite in active mode	0.0233	0.01851	0.0184	0.00862
**All participants**	**M**	**SD**	**M**	**SD**
Free walking	0.0465	0.0220	0.0447	0.0120
Suite in passive mode	0.0842	0.0421	0.0782	0.01506
Suite in active mode	0.0250	0.0110	0.0173	0.00667

**Table 5:** Pairwise comparisons of mean RMS across different modes.

Mode		MD(mV)	SE(mV)	p<0.05
Free walking	(vs) Active	0.024	0.004	0.000
Passive	(vs) Active	0.060	0.006	0.000
Free walking	(vs) Passive	-0.036	0.004	0.000

**Table 6:** Muscle activity reduction during level walking.

	Muscle Activity Reduction (%)*				
Muscle	Participant 1	Participant 2	Participant 3	Participant 4	Avg.
R. Vastus Intermedius	58.9%	59.7%	39.3%	15.6%	43.4%
R. Gastrocnemius	77.6%	64.3%	42.4%	57.1%	60.4%

* Muscle Activity Reduction(%) = (*rmsEMG*_*free walking*_ − *rmsEMG*_*active mode*_)/*rmsEMG*_*free walking*_ x 100.

Overall, the mean difference in muscle activation between the free walking mode (mode 1) and the active mode (mode 3) for all participants was found to be statistically significant at 95% confidence interval for difference: **MD** = 0.024mV, **SE** = 0.004mV, **p**<0.05 (see, Table 5). This shows net positive reduction in muscle activity on the two muscles during the active mode as can be seen also from Table 6 (Right Vastus intermedius − 43.4% and Right Gastrocnemius − 60.4%). The mean difference between the passive mode (mode 2) and the active mode is also statistically significant at 95% confidence interval for difference (**MD** = 0.060mV, **SE** = 0.006mV, **p**<0.05) indicating reduction in muscle activity. Between the free walking mode and the passive mode, the difference is also statistically significant (**MD** = -0.036mV, **SE** = 0.004mV, **p**<0.05) indicating however a net increase in muscle activation level.

### The DUKF robustness analysis: Simulations and validation

The extensive Monte Carlo simulations [62, 63] was applied to test (and validate) the robustness of the constructed DUKF for states generation before deployment on the actual prototype exoskeleton. These simulations test the variations in the process and measurement noise realization, the initial states guess, and states covariance guesses. Generally, the key signal of interest for validating the states estimation/generation is the residuals (or innovations), ${\tilde{y}}_{k}=y_{k}-{\hat{y}}_{k}^{-})$, which should satisfy three criteria:

1. Should have small magnitude
2. Zero mean and
3. No autocorrelation, except at zero lag.

**Fig 11:**
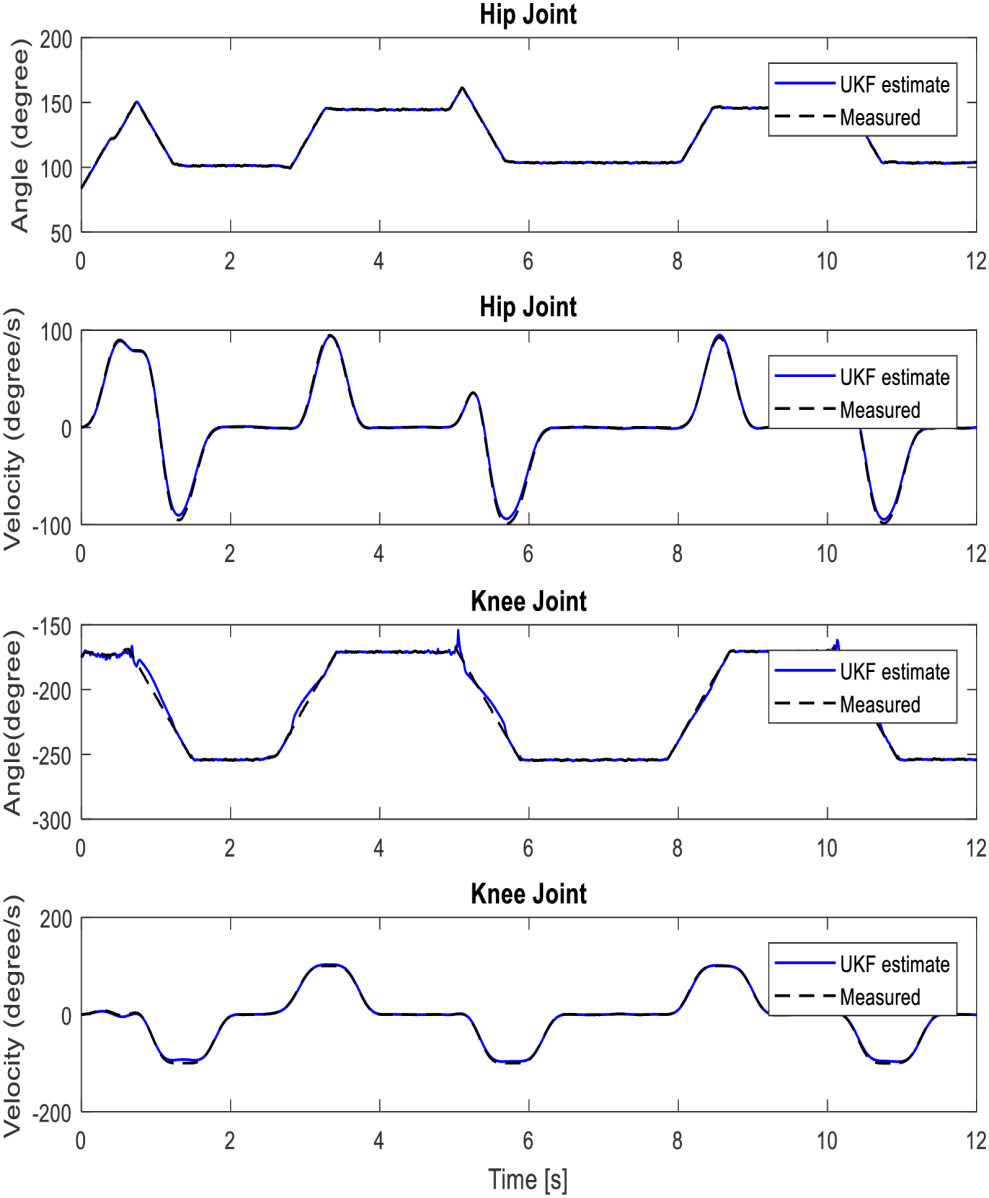
UKF simulation performance: Comparison between generated states and measured states.

**Fig 12:**
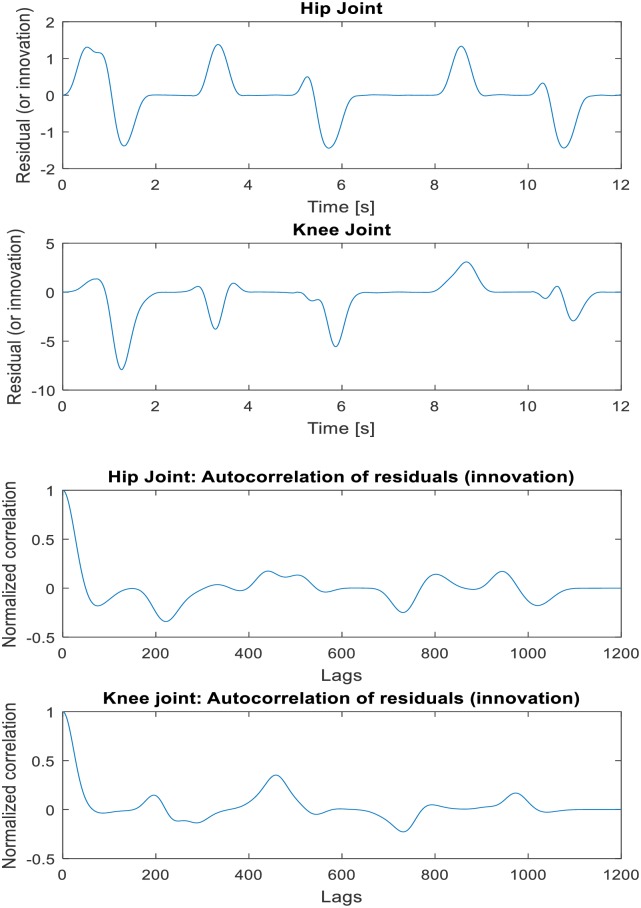
The residuals of the innovation and normalized autocorrelation of the residuals.

Prior to validating the robustness of the DUKF for state estimation/generation, we make our initial guess of states and covariance matrices. For both simulation and experiment, our initial states guess is given as **x**_*k*_ = [84, −175, 0, 0, 0, 0]^*T*^; where the first three elements (joint angles, *q*^*T*^) are taken experimentally from the position sensors on the hip, knee, and ankle in double stance phase, and the last three elements (joint velocities,${\dot{q}}^{T}$) are arbitrarily assumed zero since the velocities are small in this phase. Our initial guess of process noise covariance is specified as **v** = *diag*([0.8, 1, 1, 1, 1, 1]) to account for model inaccuracies and the effect of unknown disturbances on the plant. The higher values of process noise covariance reflect the knowledge that the states are more impacted by modelling errors. We also provided our knowledge (initial guess) of (sensor) measurement noise covariance as **n** = *diag*([0.8, 0.8, 0.8, 0.8, 0.8, 0.8]). The DUKF generated states (simulation results) using recorded hip and knee gait trajectory as sensors’ input to the filter is shown in Fig 11. The mean values of the residuals are found to be 0.0229 degrees and -0.4968 degrees respectively, which are small relative to the magnitude of the residuals, indicating no divergence in states estimation, and good filter performance. See Fig 12 for a plot of the residuals and the autocorrelation of the residuals. The mean correlations of the residuals are also close to zero (0.0011 for hip and 0.0362 for knee). They are found to be small for all lags except 0 and does not show any significant non-random variations. Ideally, the mean correlation of residuals is also required to be small, zero mean, and uncorrelated with less variance within filter error covariance estimate. These characteristics increase the confidence in filter performance and an indication of the robustness of the filter. Refer to Table 7 for summary of the filter performance.

**Table 7:** Summary of DUKF performance/robustness analysis.

Joint	Mean Value of Residuals (degrees)	Mean correlation of Residuals (degrees)	Mean states estimation error (degrees)	1-sigma uncertainty bounds(%)
**Hip**	0.0229	0.0011	State 1: -0.7490	State 1: 0
			State 2: -0.7490	State 2: 39.72
**Knee**	-0.4968	0.0362	State 1: -0.5792	State 1: 27.81
			State 2: -0.5792	State 2: 46.29

**Fig 13:**
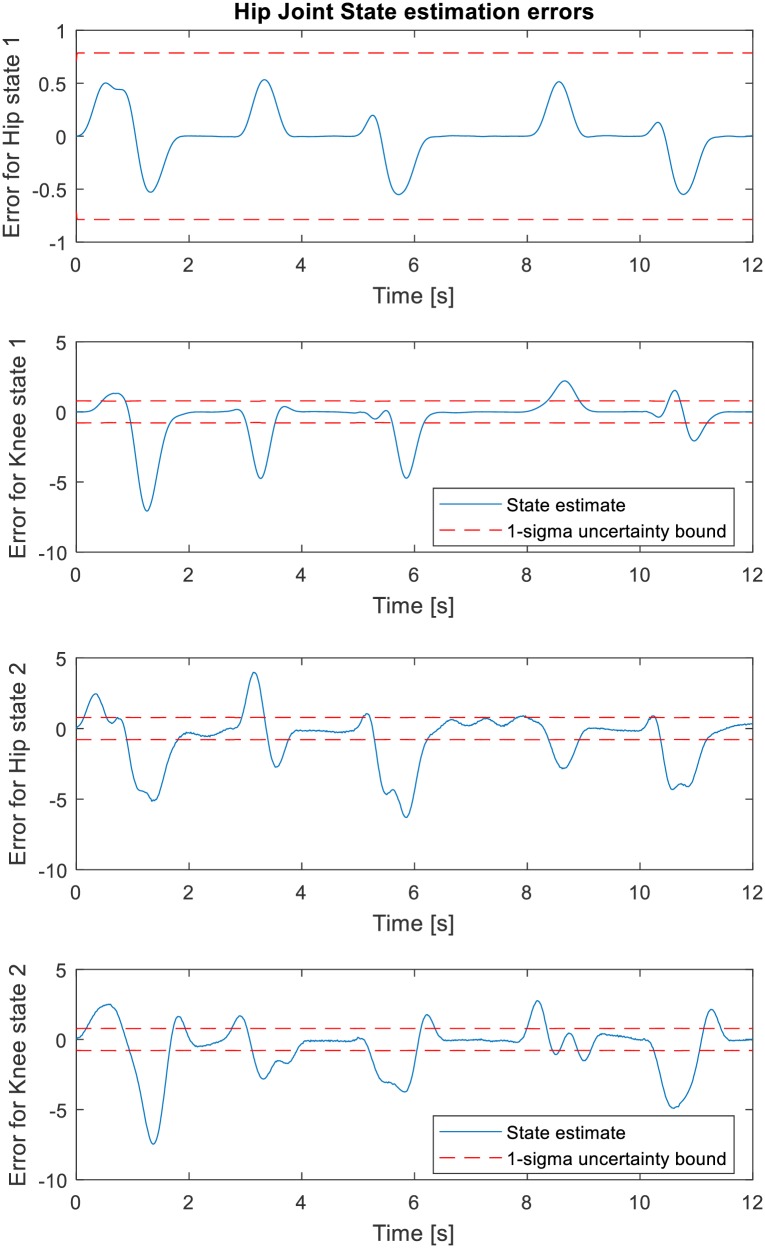
The states estimation error and the 1-*σ* uncertainty bound from the filter error covariance estimate.

**Fig 14:**
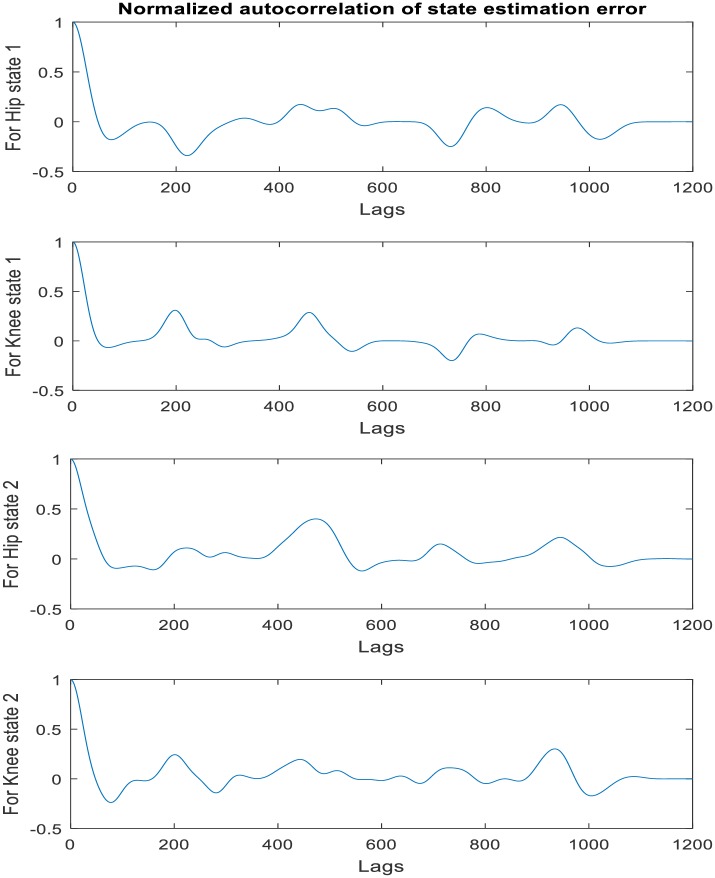
The normalized autocorrelation of states estimation error.

The error between the estimated states ${\hat{x}}_{k}$ and the true states **x**_*k*_ (just as with the residuals) is also found to be small and uncorrelated with approximately zero mean, indicating boundedness of the states’ estimation errors. Fig 13 shows the states estimation error and the 1 − *σ* uncertainty bounds from the filter error covariance estimate. The *σ* (sigma) uncertainty bounds indicate the confidence interval around the best estimate. Less than 30% of the errors exceeding the 1-sigma uncertainty bound implies good estimation. The first states estimation (i.e. joint positions) errors for hip and knee exceed the 1-sigma uncertainty bound by approximately 0 percent and 27.81% (less than 30%) respectively of the time steps which indicate good confidence and robustness in the filter performance. The second states estimation (i.e. joint velocities) are slightly higher than 30% (Refer to Table 7). However, the mean values of the errors are small relative to the value of states which suggest overall confidence in filter performance. Fig 14 shows the autocorrelation plot of state estimation error which give little non-random variations for small lag values.

### Joint friction and stiffness torque estimation

**Fig 15:**
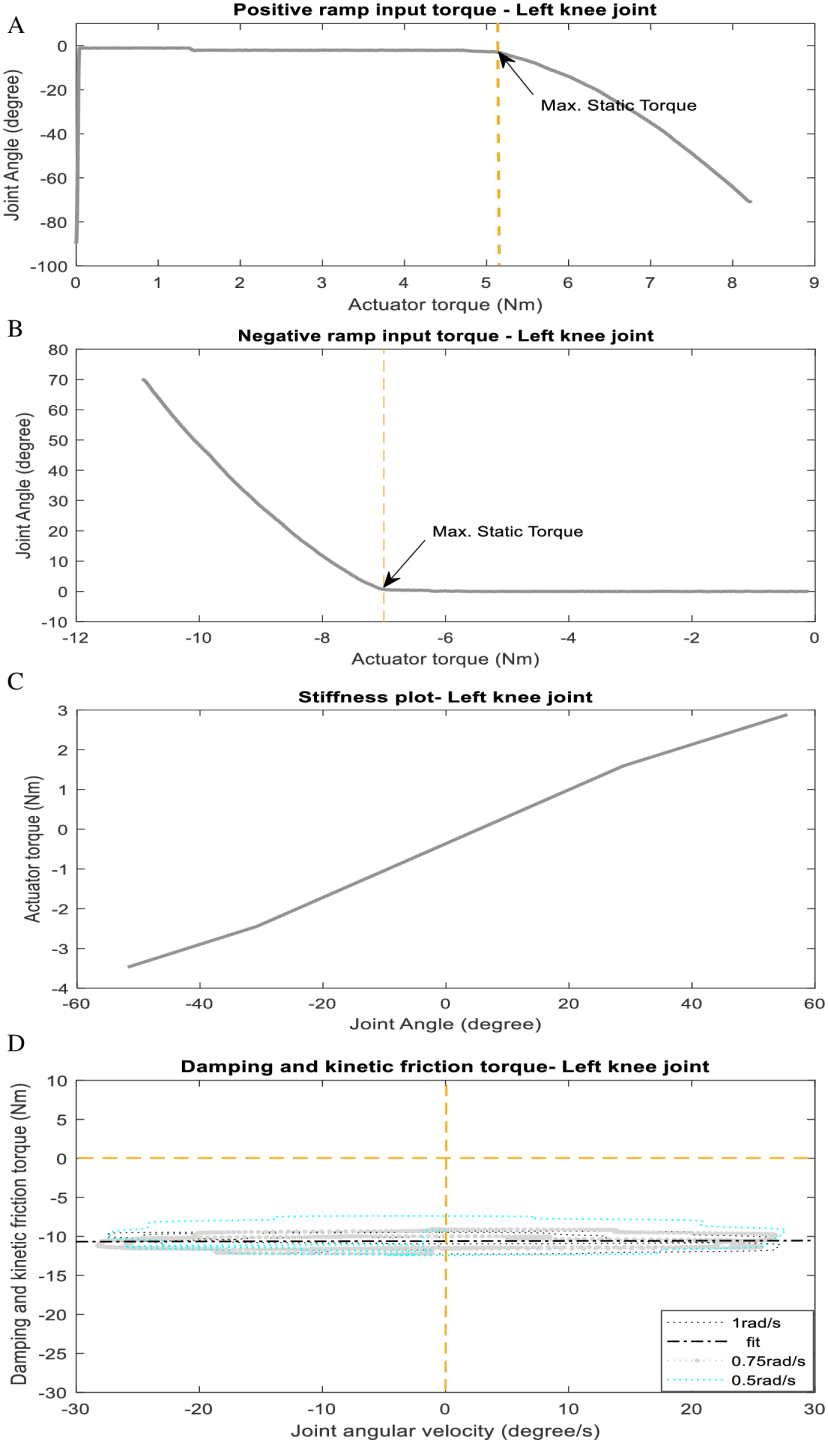
Friction and stiffness torque estimation.

We estimated the parameters of the friction and stiffness torque experimentally using a combination of static $\left(\dot{q}=0,\ddot{q}=0\right)$ and dynamic $\left(\dot{q}\neq 0,\ddot{q}\neq 0\right)$ experiments. For analysis, the actual joint angles and torques are measured from joint angle sensors and torque sensor respectively. In the static experiment, the exoskeleton joints (active hip and knee DoF) are controlled to fixed positions. The kinetic and damping friction are thus zero. We operated each joint in a closed-loop current (or torque) control mode such that the joints can be driven by a reference torque (or current) value. The static friction torques for the hip and knee joint are estimated by driving a positive and negative ramping signal (see Fig 15A and 15B) and locating the threshold where the joints begins to move. We compute the average of the two signals as the static friction torque (Table 8). The stiffness torque on the other hand is estimated by driving the joints to fixed static positions around the zero reference angles using a simple proportional controller *u* = −*K*_*p*_(*q* − *q*_*d*_) where *u* is the voltage sent to the actuators, *q*_*d*_ is the desired joint angles and *K*_*p*_ is the proportional gain. The stiffness torque parameters are then computed from a regression plot of actuating torques versus static joint positions, after subtracting the effect of static friction torque and gravitational torque, see Fig 15C.

**Table 8:** Summary of joint friction and stiffness torque estimates.

Torque	Left Hip Joint	Left Knee Joint	Right Hip Joint	Right Knee Joint
**Avg. Static Friction**	*b*_0_ = 3.830Nm (0.06A*)	*b*_0_ = 6.035Nm (0.074A*)	*b*_0_ = 2.130Nm (0.045A*)	*b*_0_ = 5.318Nm (0.065A*)
**Stiffness torque**	*τ*_*s*_(*q*) = (0.20Nm/deg)*q* − 0.18Nm	*τ*_*s*_(*q*) = (0.061Nm/deg)*q* − 0.39Nm	*τ*_*s*_(*q*) = (0.31Nm/deg)*q* − 0.042Nm	*τ*_*s*_(*q*) = (0.015Nm/deg)*q* − 0.058Nm
**Damping/kinetic friction**	$b_{2}\left(\dot{q}\right)=\left(-21.909\right)\dot{q}\text{Nm}$	$b_{2}\left(\dot{q}\right)=\left(-10.609\right)\dot{q}\text{Nm}$	$b_{2}\left(\dot{q}\right)=\left(-28.009\right)\dot{q}\text{Nm}$	$b_{2}\left(\dot{q}\right)=\left(-6.7238\right)\dot{q}\text{Nm}$

* Actuator current at static torque.

In the dynamic experiment, the joints are driven on a reference trajectory by same proportional controller *u* = −*K*_*p*_(*q* − *q*_*d*_). The joint static friction torques are zero in this case since the joints are in motion. The joint kinetic and damping friction torque are computed by a regression analysis of actuating torques versus joint angular velocities (from motor rpm) after subtracting the effect of stiffness and gravitational torques (Fig 15D). Our estimate of kinetic (or coulomb) friction torque in this experiment is zero, only the damping friction torques are significant. Table 8 presents summary of the joint stiffness and friction estimates.

### Gait prediction and trajectory tracking: Experiment

**Fig 16:**
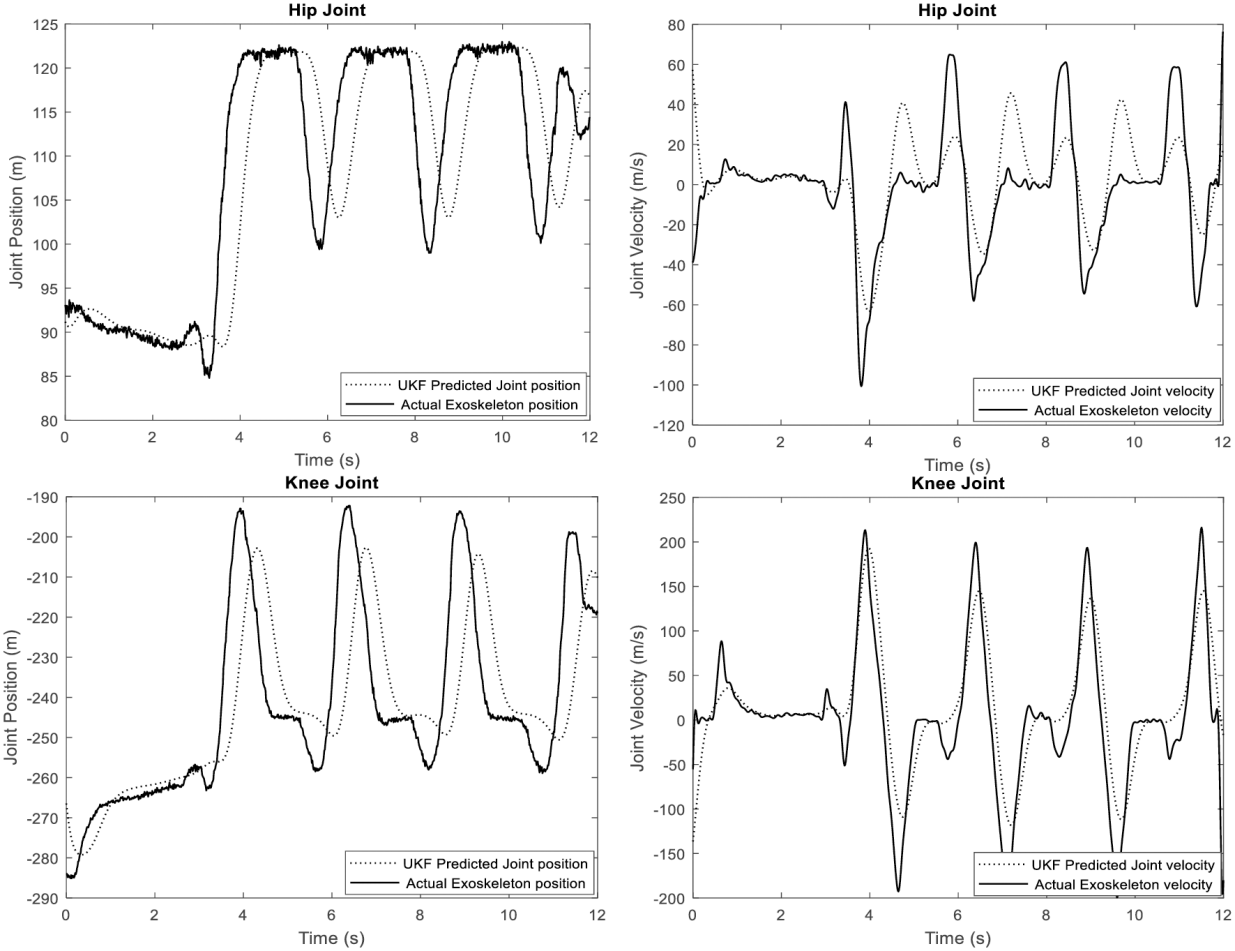
Generated left hip and left knee walking trajectory for participant 1.

The DUKF real time predicted wearers’ (hip and knee) joint trajectory from joint position sensors serves as the reference trajectory to the controller during the walking experiment. Fig 16 shows the generated walking trajectory. For the sake of brevity, we present performance for the left knee and left hip respectively for one participant. Fig 16 also present a comparison between the predicted gait and the actual exoskeletons movement. The exoskeleton system is seen to achieve synchronous walking with the participant. Table 9 gives the root mean square error of the motion tracking.

**Table 9:** Trajectory tracking performance.

Joint	Position Trajectory RMSE (degrees)	Angular Velocity Trajectory RMSE (degrees/s)
**Hip**	0.0201	0.0849
**Knee**	0.2420	0.3181

### Online parameter estimation

**Fig 17:**
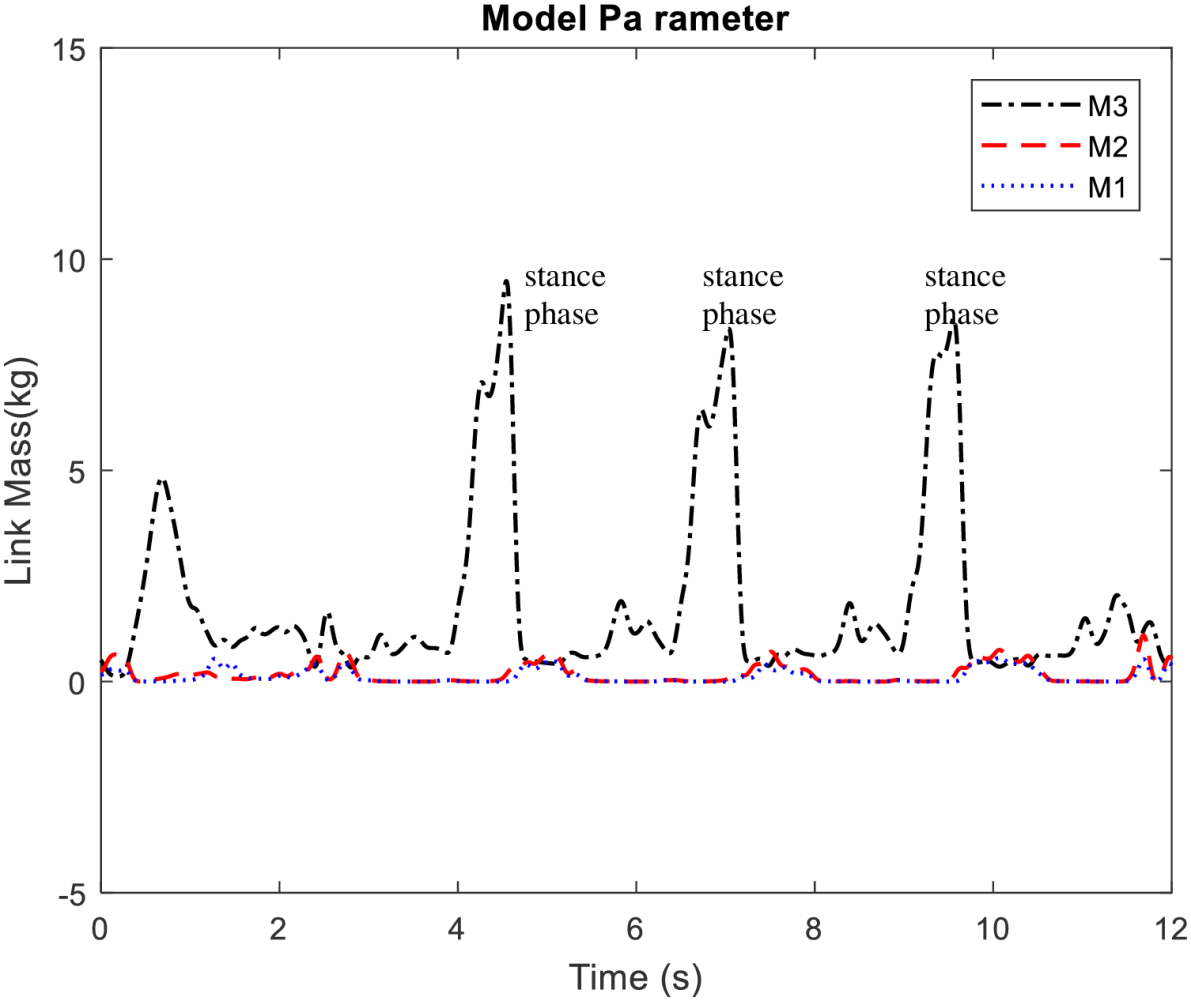
Estimated model parameters during walking movement.

The DUKF online estimated model parameters are shown in Fig 17. The model parameters are the link masses: *m*_1_, *m*_2_, and *m*_3_. The parameter *m*_3_ (i.e. the mass of thigh) is influenced by the torso mass during the stance phase. More parameter variations are thus noticed for *m*_3_ during stance to swing transition. Parameter variations for the other links are somewhat straight.

## Discussion

The results of the experiment show that the exoskeleton control method can be applied for walking assistance in prolong walking conditions. There is significant reduction in the level of muscle activation recorded at the two muscles (on the right leg) when assisted with the exoskeleton. On the average, 43.4% (Right Vastus intermedius) and 60.4% (Right Gastrocnemius) reductions in muscle activity (Table 6) was found across the participants during active assistance (compared to free walking) which gives indication of its potential benefit and health saving if the technology is applied to more than one workplace personnel. For all participants, the percent reduction in muscle activity are comparatively significant (Participant 1: Right Vastus intermedius = 58.9%, Right Gastrocnemius = 77.6%; Participant 2: Right Vastus intermedius = 59.7%, Right Gastrocnemius = 64.3%; Participant 3: Right Vastus intermedius = 39.3%, Right Gastrocnemius = 42.4%; and Participant 4: Right Vastus intermedius = 15.6%, Right Gastrocnemius = 57.1%), see Table 6. Reduction in muscle activity from other connected muscles (not monitored in the experiment) for walking is also expected.

In passive mode, the burden of the exoskeleton on the wearer is apparent. More muscle activity was required by the user to walk with the exoskeleton during the swing phase (Fig 9). The intrinsic rotational stiffness from the unactuated motor bearings may have accounted for the resistance to the wearers’ swinging leg. This fact has been noted for future design improvement. In stance phase, the contact with the ground minimizes this burden since the exoskeleton’s weight is driven to the ground through the foot contact and also since little movement is needed for stance. The burden of the exoskeleton on the wearer in both passive and active mode is generally not required to be significant so as not to dissuade acceptability of the suite in industries. The findings from this implementation suggests the need for appropriate choice of materials and motor characteristics in the design of exoskeleton suites.

With regards trajectory generation, the performance of the controller is evident from results in Fig 11, Fig 16, and Table 9. The dual UKF demonstrated capability for trajectory generation and estimation of the unobserved states i.e. the spatio-temporal features (i.e. joint position, velocity and acceleration) of the human walking. These figures compare favorably with some generated gait trajectories found in literatures [52, 53]. Similar to many other applications of Kalman filter, for experiment, the filter relies on the signal (noisy) from position sensors installed at the hip and knee flexion/extension rotational axis; and on the partially estimated model of the human-exoskeleton system. The predicted hip and knee joint angle and velocity trajectories are also compared with the actual exoskeleton movement as shown in Fig 16. The exoskeleton (actual) velocity for comparison purpose is calculated from the motor rpm. We found some limitation in synchronous walking (slightly high RMSE of 0.2420 degrees and 0.3181 degrees/s respectively for the knee joint angular position and velocity, see Table 9) which is adduced to the hardware response rate. The impulsiveness of the human nature and the computer communication rate are observed to have possible influence on the exoskeleton response rate.

The dual estimation approach of the UKF was also found useful in this experimentation to improve the model parameters and states estimates concurrently. There is some variation in the model mass parameter between the stance and swing phase, especially for the thigh mass. The thigh mass *m*_3_ increases significantly during the stance phase which can be ascribed to the loading effect of the torso mass on the thigh (Fig 17). Notice that variations in model mass parameters can be caused by external loads on the links in different postural configuration during stance or swing. The real time estimation of the model mass parameters thus captures these variations in the different phases. Consequently, feedback of these parameters in the exoskeleton model improves the accuracy of the model for each respective phase and ensures that the model is more accurate for states estimation.

Our current system can enable a pilot to walk at an average human (normal) walking speed (1.3m/s) while lifting and carrying a payload of 2.0kg on level ground. This is an applicable speed range for manual handling industry operations. Higher speed levels are possible however our current system has not been rigorously tested for higher speed walking. In comparison, HUALEX system by Huang, et al. [33] has been reported to support walking at human speed range of 0.4m/s to 1.2m/s using DMP algorithm, but can only attain synchronous walking after one gait cycle correction. The authors have suggested a proactive learning framework for future work. BLEEX [24] on the other hand can enable pilot walking up to the average normal human speed of 1.3m/s using SAC algorithm (while supporting a load of 34kg), however the system is unstable with SAC algorithm. Moreover, the system is highly powered relatively, and uses a harness at the back to support the heavy load. Our system does not use a harness, its application is mainly intended for lower limb locomotion in manual-hand lifting and carrying of medium size loads (<25kg) as typically done in manual handling industry works. With respect to the amount of muscle activity reduction, comparison of our system with the aforementioned exoskeleton systems could hardly be possible since limited information (in this regard) has been reported about them. Meanwhile, more experimental procedure and future work would be required for proper comparison of our system with some existing powered exoskeleton systems.

To the best of the authors knowledge, this study is a novel application of dual unscented Kalman filter (in a supervisory control framework) for trajectory generation (and control) of the dynamic human walking (typical in industrial manual handling operations) using a partial model of the coupled human-exoskeleton system and noisy signal from position sensors. Other closely related technique like the traditional adaptive frequency oscillators (AFOs) have been quite useful and popular for gait trajectory generation however they are mainly suitable for generating uniform (or pseudo-uniform) walking trajectory from sensor signal. Dynamic movement primitive (DMPs) on the other hand encompass AFOs to allow dynamic walking generation, but their robustness to dynamic interaction situation may be an issue. Moreover, they require considerable expertise knowledge or experience to derive an appropriate policy or non-linear equation to represent the dynamic human walking. EMG Feedback control are also very useful and popular however the inconsistencies in EMG estimation and the modality of application in feedback control are critical issues to consider.

## Conclusion

In the study we proposed an exoskeleton control strategy for synchronous walking assistance in manual handling works that involve prolong hours of walking. The strategy of assistance is based on real time trajectory generation of the spatio-temporal features of human walking, i.e. hip and knee joints position, and velocity, and acceleration, for control of the human-exoskeleton system. By this, we adopt the notion to movement primitive which suggests representation of dynamic movement behavior such as walking using kinematic representation. Kinematic representation of movement primitive is thought to offer more flexibility for workspace planning of complex movements than direct motor command. Our proposed strategy is an integration of dual unscented Kalman filter for trajectory generation and an impedance controller for trajectory following. To enable synchronous walking between the human and exoskeleton system we implemented a supervisory hybrid automaton which coordinate the coupled movement based on the detected human intention and walking phase.

The effectiveness of the controlled system to reduce lower limb muscle activity has been evaluated on four participants in a mimicked load carrying industrial scenario. There is more than 40% reduction in muscle activity recorded at the Right Vastus Intermedius and Gastrocnemius muscle of the participants during the walking trials. The controller is also able to synchronize the movement of the exoskeleton with that of the participants using the generated walking trajectory from the dual unscented Kalman filter. One potential limitation in the experimental setup however is the response time of the exoskeleton to the control signal which can be adduced to the communication layers from the host model on Simulink to the exoskeleton actuation system. The experimental findings presented in this study have nonetheless indicated that this strategy could be deployed to benefit workers engaged in prolong walking in dynamic working environment where movement behaviour may be non-uniform and complex.

## Supporting information

S1 Data Minimal dataset. The dataset is in .sav format (SPSS) and consist of EMG signal values taken from two muscle groups (of each participant): R. Vastus Intermedius and R. Gastrocnemius, in three walking trials and three mode of exoskeleton assistance.
(RAR)
